# A colour image encryption method based on a coupled map lattice–elementary cellular automaton neurodynamic chaotic system and dynamic dual-affine substitution-box architecture

**DOI:** 10.1098/rsos.260887

**Published:** 2026-09-09

**Authors:** Xin Xie, Sheng Yang, Hao Ning, Yu Zhou, Kun Zhang

**Affiliations:** School of Information Science and Technology, Hainan Normal University, Haikou, People's Republic of China

**Keywords:** colour image encryption, spatio-temporal chaos, coupled map lattice, elementary cellular automata, Wilson–Cowan model

## Abstract

Secure colour image transmission requires encryption schemes in which randomness generation, nonlinear substitution and diffusion are designed as a coherent whole. Conventional coupled map lattice-based methods are often constrained by fixed neighbourhood evolution and limited coupling diversity, while many chaos-based substitution-box (S-box) schemes use chaotic sequences mainly as external shuffling sources. This article develops a colour image encryption method that couples adaptive spatio-temporal chaos with dynamic finite-field substitution and coordinated permutation–diffusion. In the proposed chaotic model, lattice interactions and local evolution are regulated by the system state, allowing the generated sequences to exhibit stronger complexity, sensitivity and distribution uniformity. The resulting chaotic flow is further embedded into a dual-affine construction over GF(2^8^), producing key-dependent bijective S-boxes with enhanced nonlinear substitution capability. For image encryption, time-varying bit-switching transform (TV-BST) permutation, Hopfield-driven time-varying coupled map lattice (HDTCML) block key mixing and dynamic S-box-based bidirectional diffusion are integrated to jointly strengthen global scrambling, local confusion and plaintext sensitivity. Experimental analyses show that the proposed scheme produces random-like ciphertexts, suppresses adjacent-pixel correlation and improves resistance to statistical analysis, differential attacks and chosen-plaintext attacks.

## 1. Introduction

With the rapid development of cloud services, intelligent terminals and networked vision systems, colour images are increasingly captured, transmitted and stored in open environments. Since such images may contain identity-related, medical, commercial or surveillance information, protecting their confidentiality has become an important issue in multi-media security [1,2].

Unlike textual data, images have strong pixel correlation, non-uniform intensity distributions and inter-channel dependency. Directly applying conventional block ciphers may fail to sufficiently hide these statistical features and may also reduce processing efficiency [3]. Therefore, image encryption schemes should jointly consider key randomness, spatial decorrelation, nonlinear confusion and diffusion propagation to meet the security requirements of colour image protection [4].

Among the existing approaches, chaos-based cryptography has become an important branch of image encryption because nonlinear chaotic systems can provide high sensitivity to initial states and control parameters, together with pseudorandom and ergodic behaviours [5,6]. These characteristics are particularly useful for deriving keystreams, permutation indices and diffusion masks in permutation–diffusion-oriented image ciphers [7]. In early studies, the chaotic source was usually constructed from low-dimensional maps, such as logistic, tent, sine maps or their simple cascaded variants. Pak & Huang, for example, generated a keystream by exploiting the difference between two isomorphic one-dimensional chaotic branches and embedded it into a linear–nonlinear–linear encryption structure [8]. However, such low-dimensional systems are strongly affected by finite-precision implementation. Their trajectories may gradually collapse into short cycles or periodic windows, which weakens the effective key space and reduces the unpredictability of the generated sequence [9–11]. Therefore, relying only on simple low-dimensional maps is usually inadequate for high-resolution colour image encryption, where long keystreams, strong decorrelation and high plaintext sensitivity are simultaneously required.

To improve the dynamic complexity of chaotic sources, later encryption schemes have gradually moved from single-map structures to hyperchaotic systems, multi-scroll attractors, fractional-order models and memory-related chaotic systems [12]. Meanwhile, parameter perturbation, map reconstruction and hybrid coupling strategies have also been introduced to enlarge the chaotic region and enhance key sensitivity [13,14]. Subramani *et al.* [15] applied current-modulated edge-emitting semiconductor lasers to image encryption and showed that laser-based chaotic signals can support secure image encryption and security analysis. Yang *et al.* [16] constructed a class of discrete memristive hyperchaotic maps with multi-cavity and multi-structure attractors, showing their potential for secure communication. Akmeşe *et al.* [17] investigated time-series classification of discrete-time chaotic systems using deep learning approaches, demonstrating that neural models can characterize and classify chaotic sequence behaviour. These works suggest that strengthening the chaotic generator is necessary. Nevertheless, a more complex chaotic source does not automatically lead to a more effective cipher unless its output is tightly matched with the substitution, permutation and diffusion stages.

Following this direction, spatio-temporal chaotic models have been increasingly introduced into image encryption, including coupled map lattices, cellular automata and small-world-network-based systems [18]. Different from isolated low-dimensional maps, these models evolve through local interactions and parallel lattice updates, making them more suitable for producing large-scale control sequences for image-level encryption. For example, Yogi & Khan proposed a hybrid chaotic and cellular-automata-based image encryption scheme for Internet of Things (IoT) and resource-constrained devices, showing that CA-driven lightweight dynamics can improve security while maintaining implementation efficiency [19]. However, most existing spatio-temporal chaos-based schemes still use the generated chaotic sequence mainly as a numerical keystream. The internal relationship between spatio-temporal evolution, nonlinear substitution and the final permutation–diffusion architecture is often not sufficiently established.

In addition to the chaotic source, the substitution-box (S-box) is another key component affecting the confusion capability of an encryption system. As a nonlinear substitution mapping, it plays an important role in resisting linear and differential cryptanalysis [20,21]. Traditional S-boxes are commonly designed from finite-field algebraic structures, such as power mappings, affine transformations, bent functions and differentially uniform functions. These constructions have clear mathematical foundations, but they are usually fixed once generated and are not easily associated with the secret key, the plaintext state or the dynamic evolution of the encryption process. To improve S-box quality, researchers have introduced heuristic and intelligent optimization methods that jointly consider nonlinearity, differential uniformity, linear approximation probability and the strict avalanche criterion. For example, Jiang & Ding [22] combined bent functions with chaotic sequences to construct a hybrid substitution mechanism. Wang *et al.* [23] adopted a genetic algorithm to optimize bijective S-boxes and obtained improved cryptographic indicators compared with basic constructions. Alhadawi *et al.* [24] combined discrete chaotic maps with cuckoo search to generate competitive S-boxes under relatively low parameter complexity.

In recent years, chaotic-sequence-driven S-box construction has become a common strategy in image encryption. Typically, a real-valued chaotic sequence is quantized and then used to shuffle or permute the 0–255 index set, so that a key-related or plaintext-related S-box can be obtained [25,26]. Compared with fixed substitution tables, this type of dynamic S-box can improve local confusion and increase the adaptability of the encryption process [27]. Representative examples include the dynamic key-related S-box constructed by Liu *et al.* using a three-dimensional chaotic map [28], the Arnold-map-driven dynamic S-box generation method proposed by Luo *et al.* [26] and the fractal–chaos hybrid S-box designed by Ge & Li [29]. Nevertheless, in many existing schemes, the chaotic system only provides random-like numbers for index shuffling. The S-box generation process itself is not deeply coupled with the spatio-temporal structure of the chaotic system, and its interaction with the subsequent image-level permutation and diffusion operations remains limited.

Based on the above analysis, several issues still need to be addressed. First, many existing methods enhance the chaotic generator and the image encryption procedure separately, but do not sufficiently consider whether the generated dynamics can continuously support substitution and diffusion under finite-precision computation. Second, although many chaotic S-boxes are dynamic in form, their construction is often driven by discretized chaotic values only, rather than by the structural evolution of the underlying spatio-temporal system. Third, for colour image encryption, global spatial scrambling, channel-level diffusion and key-dependent nonlinear substitution are still often designed as loosely connected modules, which limits the cooperation between local confusion and global diffusion. Therefore, it is necessary to construct a unified encryption framework in which the chaotic source, S-box construction and permutation–diffusion architecture are jointly designed.

To address these problems, this article proposes a colour image encryption framework that integrates a coupled map lattice (CML)–elementary cellular automaton (ECA) neurodynamic chaotic system with a time-varying bit-switching transform (TV-BST) architecture. The proposed framework is designed to connect the dynamic source with the cryptographic components and the image-level encryption process. Specifically, the CML–ECA neurodynamic system provides spatio-temporal chaotic sequences through ECA-based structural regulation and neurodynamic feedback; the generated chaotic flow is further used to construct and optimize a dynamic S-box; and the TV-BST architecture combines key-dependent row–column permutation with channel-wise bidirectional diffusion. The main contributions are summarized as follows.

— A hybrid adaptive spatio-temporal chaotic system is proposed for cryptographic sequence generation. To address the fixed neighbourhood structure, single coupling mode and limited sequence complexity of conventional CML systems, ECA dynamic states and Wilson–Cowan excitatory-inhibitory feedback are introduced so that lattice-neighbourhood selection, local control parameters and coupling strength can adaptively vary with the system state.
— A dynamic finite-field dual-affine S-box generation method driven by the proposed chaotic system is designed. The chaotic sequence dynamically determines the irreducible polynomial, affine matrices and offset vectors over GF(2^8^), producing key-dependent bijective S-boxes with improved nonlinearity and enhanced resistance to differential analysis.
— A colour image encryption framework combining TV-BST permutation, Hopfield-driven time-varying coupled map lattice (HDTCML) block key mixing and dynamic S-box-based bidirectional diffusion is constructed. The framework coordinates global scrambling, nonlinear substitution and feedback diffusion, resulting in lower pixel correlation, higher entropy, near-ideal number of pixels change rate (NPCR)/unified average changing intensity (UACI) values and stronger resistance to statistical, differential and chosen-plaintext attacks.

The remainder of this article is organized as follows. Section 2 reviews the related foundations, including ECA, two-dimensional CML and S-box. Section 3 presents the proposed CML–ECA neurodynamic chaotic system and its dynamical analysis. Section 4 describes the complete encryption architecture, including TV-BST permutation, chaotic S-box generation and bidirectional diffusion. Section 5 reports the experimental results and security analyses. Finally, §6 concludes this article.

## 2. Related work

### 2.1. Elementary cellular automata

Within the framework of discrete dynamics, a one-dimensional ECA is a binary system evolving on a ring lattice of length *n*: let the state of the *i*th cell at time (*t*) be denoted by $s_{i}^{\left(t\right)}\in \{0,1\}$. Under periodic boundary conditions, its synchronous updating is defined by a ternary-neighbourhood Boolean mapping.

(2.1) $$s_{i}^{\left(t+1\right)}=f\left(s_{i-1}^{\left(t\right)},s_{i}^{\left(t\right)},s_{i+1}^{\left(t\right)}\right),i=1,\ldots ,n.$$

Consequently, a global transformation *F_R_*: {0, 1}^*n*^ → {0, 1}^*n*^ is induced to satisfy **s**^(*t* + 1)^ = *F_R_*(**s**^(*t*)^). Since the ternary neighbourhood admits 2^3^ = 8 possible inputs, any ECA rule can be uniquely specified by an 8-bit output vector **r** = (*r*_7_,*r*_6_,…, *r*_0_) ∈ {0, 1}^8^, and is encoded in the prescribed order (e.g. from 111 to 000) as

(2.2) $$R=\sum_{k=0}^{7}r_{k}\cdot 2^{k},\;\;R\in \left\{0,\ldots ,255\right\}.$$

**Figure 1.**
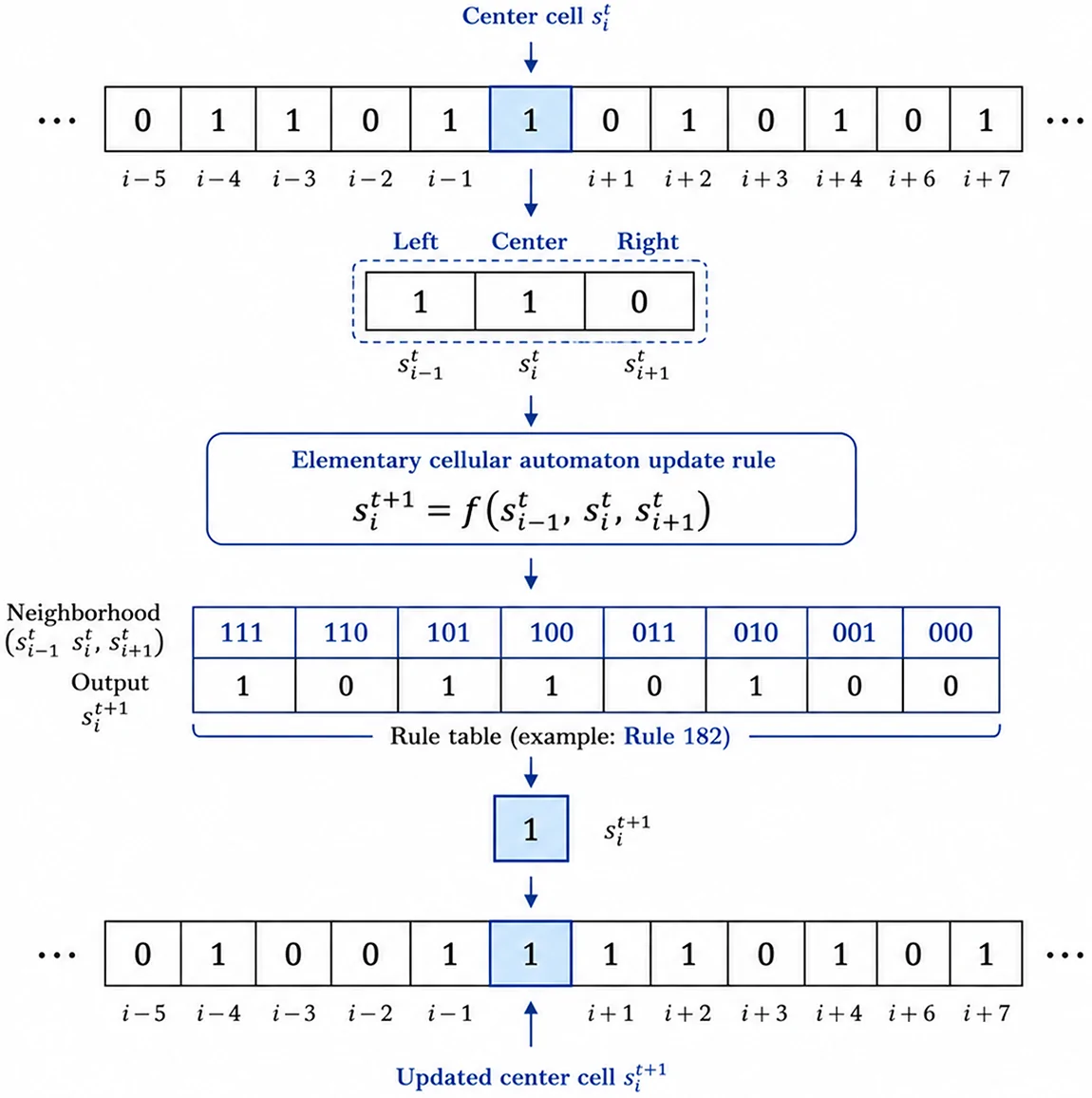
Illustration of the elementary cellular automaton update process.

Thus, 256 distinct rules are obtained. The finite state space implies that each trajectory exhibits ‘pre periodic’ behaviour: there exist *T* and *P* ∈ *N* such that *s*^(*t* + *p*)^ = *s*^(*t*)^ holds for all *t* ≥ *T*. The global state can be treated as nodes, and *F_R_* as directed edges, thereby forming a state-transition graph that characterizes attractors (cycle loops) and their basins. The update process of the elementary cellular automaton is illustrated in figure 1. For a given centre cell, its next state is determined by the current states of its left, centre and right neighbouring cells according to the selected rule table.

ECA computation involves only Boolean logic over a constant number of neighbourhood bits; hence, it offers high parallelism and low implementation cost, making it suitable as a bit-level nonlinear primitive in cryptography and image processing. By binding the rule index *R*, the initial configuration *s*^(0)^ and the number of iterations *t* to the secret key, the resulting 8-bit state sequence obtained by iteration can serve as a mask or as a substitution driver; for example, for byte *b* ∈ {0, 1}^8^, define

(2.3) $$b^{\prime }=b⊕\overset{t}{\underset{\tau =1}{⊕}}state\left(\tau \right),\;\;state\left(\tau \right)\in {\left\{0,1\right\}}^{8}.$$

This enables bit-level confusion and diffusion with an interpretable structure, tuneable parameters and hardware-friendly implementation.

### 2.2. Two-dimensional coupled map lattice system

The two-dimensional-CML is a typical spatio-temporal chaotic model, which characterizes interactions among lattice sites through local nonlinear mappings and diffusive coupling. The system consists of a two-dimensional lattice network of *L*  ×  *L*, with periodic boundary conditions (indices computed according to modL), where the state of each lattice site is simultaneously influenced by its four neighbouring sites. For an arbitrary site (*i*, *j*), the evolution rule is written as:

(2.4) $$x_{n+1}(i,j)=\left(1-\varepsilon \right)f\left(x_{n}(i,j)\right)+\frac{\varepsilon }{4}\left[f\left(x_{n}(i+1,j)\right)+f\left(x_{n}(i-1,j)\right)+f\left(x_{n}(i,j+1)\right)+f\left(x_{n}(i,j-1)\right)\right].$$

Here, *i*, *j* ∈ {1, 2, …*L*} denote the row and column indices, respectively; *n* ∈ {1, 2, …} is the time index in the iterative process; ε ∈ [0, 1] is the coupling coefficient that controls the trade-off between the local map and neighbourhood diffusion and *f*( · ) is the local mapping function. A commonly used local map is the logistic map:

(2.5) f(x)=μx(1−x).

Here, *x* ∈ [0, 1] is the state variable and  µ ∈ (0, 4] is the system control parameter. When  µ approaches 4, the local map exhibits strong chaotic behaviour. On this basis, by adjusting ε, the system can transition continuously among spatio-temporal turbulence, coherent patterns and near synchronization: when ε → 0, the lattice sites evolve almost independently; as ε increases, neighbourhood coupling is strengthened, diffusion becomes faster and synchronization or spatio-temporal structures may be induced.

### 2.3. Substitution box

The S-box is a core nonlinear component in block ciphers and various symmetric encryption structures. Its primary role is to implement a nonlinear substitution from input bits to output bits, thereby breaking linear relationships among the plaintext, key and ciphertext, and enhancing resistance against classical cryptanalysis such as linear and differential attacks. Formally, an S-box can be viewed as a mapping over a finite field from $F_{2}^{n}$ to $F_{2}^{m}$,

(2.6) $$S:F_{2}^{n}\to F_{2}^{m}.$$

Here, *n* and *m* denote the numbers of input and output bits, respectively. In typical block ciphers and image-encryption applications, an *n* = *m* = 8 S-box is commonly used to map 8-bit inputs to 8-bit outputs. This mapping is generally required to be bijective, i.e. a permutation of 0–255, which facilitates the construction of an invertible encryption–decryption process.

## 3. The chaotic system proposed in this article

### 3.1. Elementary cellular automaton-Wilson–Cowan feedback-driven chaotic system

To provide a high-complexity dynamic source for cryptographic construction, this study designs a two-dimensional adaptive coupled map lattice driven by ECA and Wilson–Cowan neural feedback. Let $X_{i,j}^{k}(t)$ denote the chaotic state at lattice position (*i*, *j*), channel *k* and iteration *t*. Unlike a conventional coupled map lattice with fixed neighbourhood interaction, the proposed system introduces time-varying ECA structures and local neural feedback to regulate both the coupling strength and the effective control parameter. This design increases the spatio-temporal uncertainty of the generated chaotic sequence while preserving deterministic reproducibility.

At each iteration, a one-dimensional ECA sequence is updated according to a dynamically selected rule from a predefined rule pool. The ECA evolution can be expressed as

(3.1) $$B_{n}(t+1)=R_{t}\left(B_{n-1}(t),B_{n}(t),B_{n+1}(t)\right),$$

where *B_n_*(*t*) ∈ {0, 1} is the binary state of the *n*th ECA cell, and $R_{t}$ denotes the rule selected at iteration *t*. The updated ECA sequence is reshaped into a two-dimensional matrix *B*_*i*,*j*_(*t*), which is then used to estimate the local structural complexity of each lattice site. For a 3  ×  3 neighbourhood, the local probability of state 1 is defined as

(3.2) $$p_{i,j}(t)=\frac{1}{9}\sum_{a=-1}^{1}\sum_{b=-1}^{1}B_{i+a,j+b}(t)$$

and the corresponding local binary entropy is

(3.3) $$H_{i,j}(t)=-p_{i,j}(t){\text{log}}_{2}p_{i,j}(t)-\left[1-p_{i,j}(t)\right]{\text{log}}_{2}\left[1-p_{i,j}(t)\right].$$

The local entropy is used to construct an adaptive coupling coefficient,

(3.4) $$\varepsilon_{i,j}(t)=clip\left(\varepsilon_{0}+\Delta \varepsilon H_{i,j}(t),\;0.03,\;0.35\right),$$

where ε_0_ is the baseline coupling strength and Δε controls the entropy-induced modulation amplitude. Thus, the coupling strength varies with the local ECA complexity instead of remaining spatially uniform.

To further enhance the adaptive behaviour of the system, a Wilson–Cowan feedback mechanism is introduced at each lattice site. The local driving term is defined by combining the current chaotic state and the local ECA entropy,

(3.5) $$D_{i,j}(t)=0.70\;{\bar{X}}_{i,j}(t)+0.30\;H_{i,j}(t).$$

The excitatory and inhibitory states are updated as

(3.6) $$\begin{array}{clc} & E_{i,j}(t+1)=E_{i,j}(t)+\frac{\Delta t}{\tau_{E}}\left[-E_{i,j}(t)+S\left(w_{EE}E_{i,j}(t)-w_{EI}I_{i,j}(t)+g_{E}D_{i,j}(t)\right)\right] & \\ & I_{i,j}(t+1)=I_{i,j}(t)+\frac{\Delta t}{\tau_{I}}\left[-I_{i,j}(t)+S\left(w_{IE}E_{i,j}(t)-w_{II}I_{i,j}(t)+g_{I}D_{i,j}(t)\right)\right] & \end{array},$$

where

(3.7) $$S(z)=\frac{1}{1+\text{exp}\left[-a(z-\theta )\right]}$$

is the sigmoid activation function. The excitatory-inhibitory balance *E*_*i*,*j*_(*t*) − *I*_*i*,*j*_(*t*) is then used to adjust the effective logistic control parameter and the local coupling coefficient,

(3.8) $$\begin{array}{clc} & u_{i,j}^{k}(t)=clip\left(u^{k}\left[1+\alpha_{u}\left(E_{i,j}(t)-I_{i,j}(t)\right)\right],\;u_{\min},\;u_{\max}\right) & \\ & {\hat{\varepsilon }}_{i,j}(t)=clip\left(\varepsilon_{i,j}(t)\left[1+\alpha_{\varepsilon }\left(E_{i,j}(t)-I_{i,j}(t)\right)\right],\;0.03,\;0.35\right) & \end{array}.$$

The chaotic state is updated by combining the local nonlinear evolution and the adaptively selected neighbourhood state,

(3.9) $$Y_{i,j}^{k}(t)=\left[1-{\hat{\varepsilon }}_{i,j}(t)\right]f_{u_{i,j}^{k}}\left(X_{i,j}^{k}(t)\right)+{\hat{\varepsilon }}_{i,j}(t)f_{u_{i,j}^{k}}\left({\bar{N}}_{i,j}^{k}(t)\right),$$

where

(3.10) $$f_{u}\left(x)=ux(1-x\right)$$

is the logistic map, and ${\bar{N}}_{i,j}^{k}(t)$ denotes the adaptive neighbourhood mean determined by the ECA structure and Wilson–Cowan feedback. Finally, a lightweight fractional whitening operation is applied,

(3.11) $$X_{i,j}^{k}(t+1)=frac\left(\phi f_{u_{i,j}^{k}}\left(Y_{i,j}^{k}(t)\right)+\gamma M_{i,j}^{k}(t)\right),$$

where *ϕ* is the golden ratio, γ is the whitening gain, $M_{i,j}^{k}(t)$ is a deterministic mask derived from the ECA cross-structure and frac( · ) returns the fractional part. Through the joint action of ECA-driven structural perturbation, Wilson–Cowan feedback modulation, adaptive coupling and fractional whitening, the proposed chaotic system produces highly sensitive and structurally complex spatio-temporal sequences suitable for subsequent cryptographic applications.

### 3.2. Adaptive neighbourhood selection and spatio-temporal coupling mechanism

**Figure 2.**
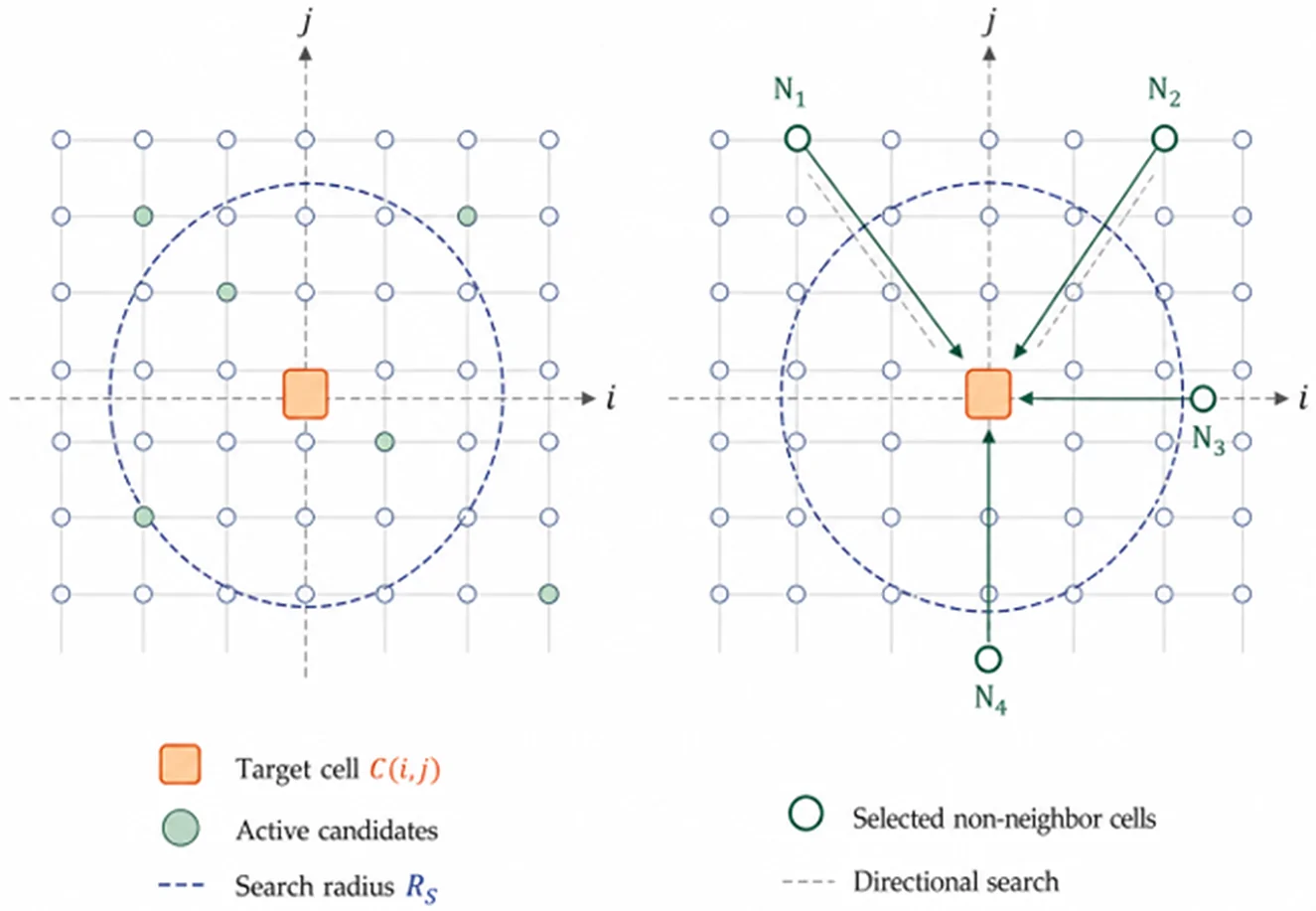
Schematic illustration of the adaptive non-neighbour selection mechanism.

In a conventional coupled map lattice, each lattice site usually interacts with a fixed set of nearest neighbours. Although such a topology is simple and easy to implement, its diffusion path remains structurally predictable. To overcome this limitation, the proposed chaotic system introduces an adaptive neighbourhood selection mechanism in which the coupled neighbours are jointly determined by the ECA field and the Wilson–Cowan feedback state. As a result, the spatial coupling path changes with both the discrete structural evolution and the local neural excitation–inhibition balance. The adaptive non-neighbour selection process is illustrated in figure 2.

For the lattice site (*i*, *j*), the candidate neighbourhood is searched under toroidal boundary conditions. Let

(3.12) D={(−1,0),(1,0),(0,−1),(0,1),(−1,−1),(−1,1),(1,−1),(1,1)}

denote the candidate directional set. A candidate neighbour can be expressed as

(3.13) $$(i^{\prime },j^{\prime })=\left(\left(i+sd_{x}\right)\;\mathrm{mod}\;N,\;\left(j+sd_{y}\right)\;\mathrm{mod}\;N\right),$$

where $(d_{x},d_{y})\in D$, *s* is the search step and *N* is the lattice size. The ECA state *B*_(*i*′,*j*′)_(*t*) acts as a structural gate for neighbour selection. A candidate site is preferentially accepted when

(3.14) $$B_{\left(i^{\prime },j^{\prime }\right)}\left(t\right)=1,$$

which means that the ECA field does not merely perturb the coupling strength, but also participates in determining the spatial information-flow path.

The Wilson–Cowan feedback further regulates the search behaviour. Let

(3.15) $$\Delta_{i,j}(t)=E_{i,j}(t)-I_{i,j}(t)$$

represent the local excitation–inhibition balance. When Δ_*i*,*j*_(*t*) > 0, the search step tends to be enlarged within the allowed radius, encouraging the system to couple with more distant lattice sites. Conversely, when Δ_*i*,*j*_(*t*) ≤ 0, the search step is reduced, making the coupling more local. This feedback-dependent search can be summarized as

(3.16) $$s_{i,j}(t)=\{\begin{array}{cllc} & \min(R,\;s_{0}+1), & \Delta_{i,j}(t)>0 & \\ & \max(1,\;s_{0}-1), & \Delta_{i,j}(t)\leq 0 & \end{array},$$

where *R* is the maximum search radius and *s*_0_ is the initial candidate step generated by the deterministic selection process. Therefore, the neighbourhood topology becomes a dynamic function of the local neural feedback state rather than a fixed geometric structure.

After four effective neighbours are selected, their channel-wise mean is calculated as

(3.17) $${\bar{N}}_{i,j}^{k}(t)=\frac{1}{4}\sum_{q=1}^{4}X_{i_{q},j_{q}}^{k}(t),$$

where (*i_q_*,*j_q_*) denotes the *qth* selected neighbour. This adaptive neighbourhood mean is then incorporated into the local nonlinear update,

(3.18) $$Y_{i,j}^{k}(t)=\left[1-{\hat{\varepsilon }}_{i,j}(t)\right]f_{u_{i,j}^{k}}\left(X_{i,j}^{k}(t)\right)+{\hat{\varepsilon }}_{i,j}(t)f_{u_{i,j}^{k}}\left({\bar{N}}_{i,j}^{k}(t)\right).$$

Here, *f_u_*(*x*) = *ux*(1 − *x*) is the logistic map, $u_{i,j}^{k}(t)$ is the feedback-adjusted control parameter and ${\hat{\varepsilon }}_{i,j}(t)$ is the feedback-adjusted local coupling coefficient. This formulation couples the self-evolution of each lattice site with a time-varying, ECA-gated and neural-feedback-modulated neighbourhood state.

The proposed adaptive coupling mechanism increases the uncertainty of spatial information diffusion in two ways. First, the ECA field introduces a discrete and time-varying structural constraint, so the same lattice site may interact with different valid neighbours at different iterations. Second, the Wilson–Cowan feedback modifies the search radius according to the local excitation–inhibition balance, allowing the system to alternate between more local and more non-local coupling behaviour. This mechanism weakens the regularity of fixed-neighbourhood CML evolution and strengthens the spatio-temporal mixing ability of the chaotic system.

### 3.3. Chaotic sequence extraction and lightweight whitening strategy

After adaptive spatio-temporal coupling, the continuous chaotic states are further processed to obtain a uniformly distributed control sequence for subsequent cryptographic construction. Since the raw lattice states may still retain local structural traces from the coupled map evolution, a lightweight whitening strategy is introduced before sequence extraction. This strategy combines the nonlinear logistic response, the ECA-derived structural mask and a golden-ratio fractional transformation to enhance the dispersion of the output states.

For each lattice site (*i*, *j*), the ECA field is first used to construct a local cross-structure mask. Specifically, the binary states from the *i*th row and the *j*th column of the ECA matrix are concatenated as

(3.19) $$c_{i,j}(t)=\left[B_{i,1}(t),\;\ldots ,\;B_{i,N}(t),\;B_{1,j}(t),\;\ldots ,\;B_{N,j}(t)\right],$$

where repeated centre information can be removed to avoid redundancy. The resulting binary vector is compressed into a channel-wise mask $M_{i,j}^{k}(t)\in (0,1)$. For every group of eight binary elements, the mask value can be written as

(3.20) $$M_{i,j}^{k}(t)=\frac{1}{256}\sum_{\ell =1}^{8}2^{8-\ell }c_{\ell }^{k}(t),$$

where $c_{\ell }^{k}(t)\in \{0,1\}$ denotes the ℓth binary element assigned to channel *k*. In this way, the discrete ECA structure is converted into a deterministic fractional perturbation term.

The coupled state $Y_{i,j}^{k}(t)$ obtained from the adaptive neighbourhood mechanism is then passed through a second nonlinear logistic response,

(3.21) $$Z_{i,j}^{k}(t)=f_{u_{i,j}^{k}}\left(Y_{i,j}^{k}(t)\right),$$

where

(3.22) f(x)=ux(1−x).

The whitened chaotic state is generated by combining this nonlinear response with the ECA-derived mask,

(3.23) $$X_{i,j}^{k}(t+1)=\text{frac}\left(\phi Z_{i,j}^{k}(t)+\gamma M_{i,j}^{k}(t)\right),$$

where $\phi =(1+\sqrt{5})/2$ is the golden ratio, γ is the whitening gain and frac( · ) extracts the fractional part. The irrational scaling introduced by *ϕ* helps disturb residual periodic structures, while the ECA-derived mask injects local structural variability into each channel. Because all operations are deterministic, the same secret parameters and initial conditions still reproduce the same chaotic sequence.

After whitening, the multi-dimensional chaotic states are flattened into a one-dimensional stream,

(3.24) $$x=\text{vec}\left(X_{i,j}^{k}(t)\right).$$

To further decorrelate adjacent elements during stream extraction, each state value is mixed with its index and the key-derived initial value,

(3.25) $$\xi_{n}=frac\left(10^{6}x_{n}+n\phi +\kappa \right),$$

where *x_n_* is the *n*th flattened chaotic value, *n* is the sequence index and κ is the key-derived fractional parameter. The final sequence {ξ_*n*_} lies in (0, 1) and is used as the control stream for parameter selection in the following S-box construction stage.

This extraction strategy plays two roles. First, it converts the high-dimensional spatio-temporal chaotic states into a reproducible one-dimensional control stream. Second, it reduces the influence of residual lattice regularity by combining nonlinear remapping, ECA-based masking, golden-ratio fractional perturbation and index-dependent mixing. Therefore, the generated sequence inherits the sensitivity of the adaptive chaotic system while providing a more suitable numerical source for cryptographic parameter generation.

### 3.4. Performance analysis

#### 3.4.1. Bifurcation diagram

Bifurcation analysis was used to evaluate the global dynamical behaviour of the proposed chaotic system under different control parameters. A bifurcation diagram describes the long-term distribution of system states as a parameter varies. It therefore provides an intuitive way to identify stable regions, periodic windows and chaotic intervals. For cryptographic applications, a desirable chaotic source should exhibit broad chaotic regions, weak periodic structures and dense state coverage over the available parameter range.

**Figure 3.**
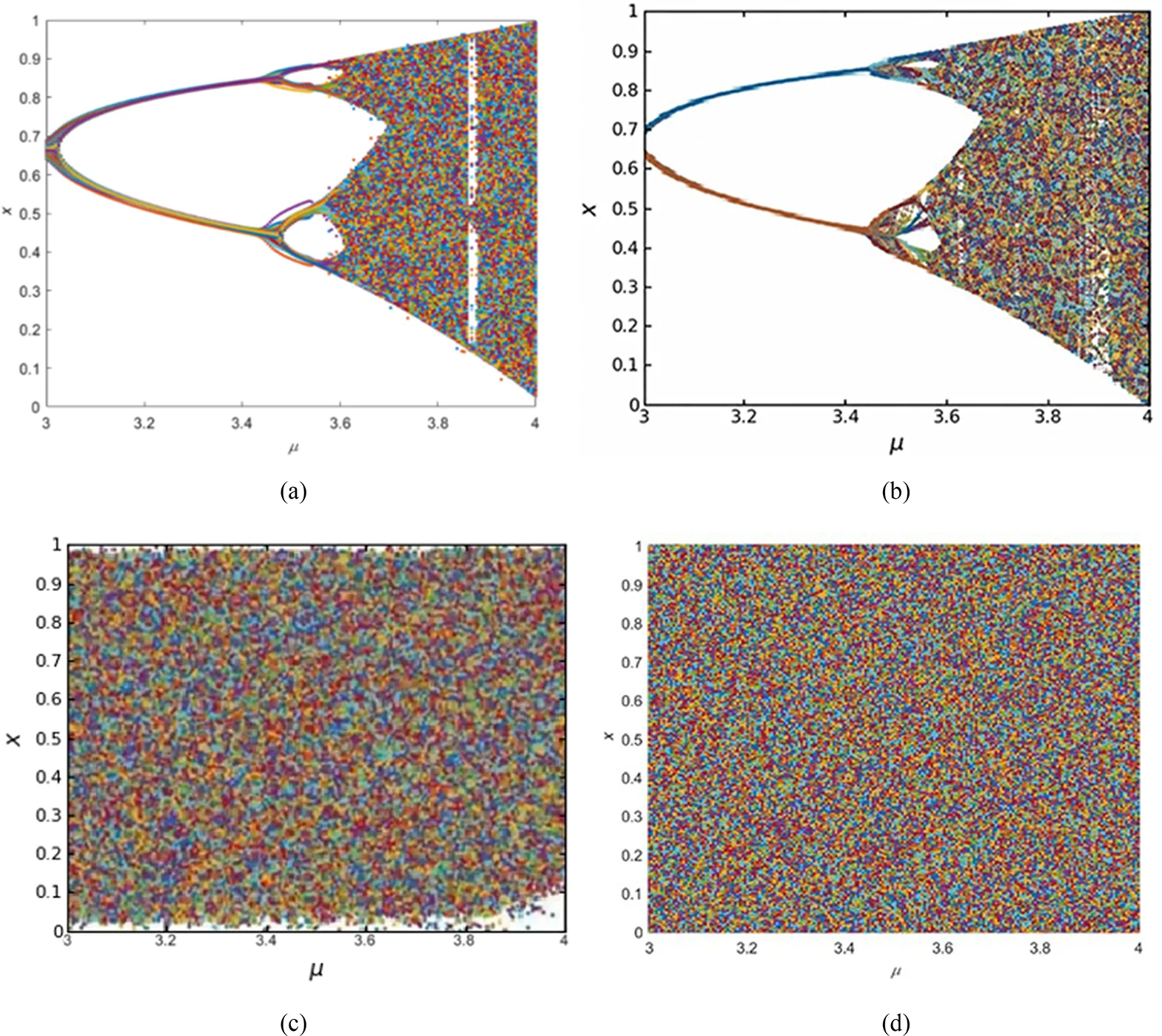
Bifurcation diagrams of different chaotic systems: (a) two-dimensional CML, (b) two-dimensional NLCML, (c) two-dimensional MCPML, and (d) the proposed system. The horizontal axis denotes the varied control parameter, and the vertical axis denotes the sampled state of lattice site (6,6) after transient iterations. A denser and more continuous distribution indicates stronger chaotic behaviour and fewer periodic windows.

Figure 3 compares the bifurcation behaviour of different two-dimensional chaotic systems by sampling the state of lattice site (6,6) after transient iterations. In the conventional two-dimensional CML, the trajectories still show evident period-doubling structures and several periodic windows, indicating that the system may enter predictable or weakly chaotic states under many parameter settings. The two-dimensional nonlinear coupled map lattice (NLCML) reduces some of these periodic branches, but local discontinuities and non-uniform state distributions remain visible. The two-dimensional mixed pseudo-random coupling piecewise linear chaotic map (MCPML) further expands the chaotic region, yet stripe-like structures and sparse local bands can still be observed, suggesting that state correlation is not fully suppressed.

By contrast, the proposed system produces a denser and more continuous state distribution across the tested parameter range. The sampled states occupy a wider portion of the available interval, and obvious periodic branches are substantially weakened. This behaviour is consistent with the combined effects of ECA-driven local entropy regulation, Wilson–Cowan feedback modulation, adaptive neighbourhood coupling and lightweight fractional whitening. These mechanisms jointly enhance spatio-temporal mixing and reduce regular lattice evolution. Therefore, the proposed chaotic system provides a more stable and less predictable dynamic source for subsequent S-box construction and image encryption.

#### 3.4.2. Lyapunov exponent and Kolmogorov–Sinai entropy

The Lyapunov exponent (LE) and Kolmogorov–Sinai (KS) entropy were further used to quantify the chaotic intensity of the system. The LE measures the average exponential divergence rate between nearby trajectories. A positive LE indicates sensitive dependence on initial conditions, which is a fundamental property of chaotic dynamics. For a one-dimensional discrete map, the LE is defined as

(3.26) $$\lambda =\underset{n\to \infty }{\lim}{\sum_{i=0}^{n-1}\text{ln}\left|\frac{dF(x)}{dx}\right|}_{x=x_{i}}.$$

**Figure 4.**
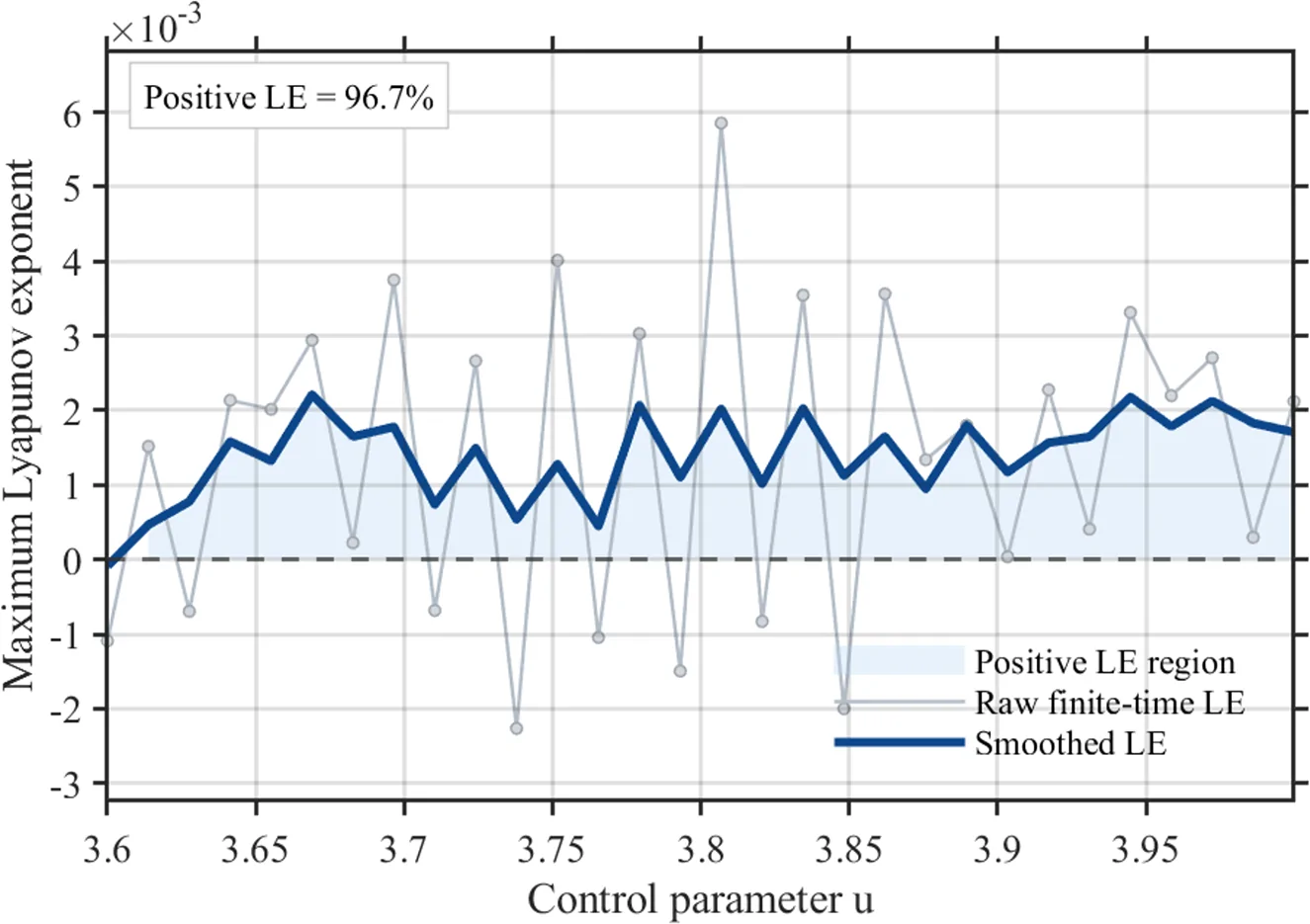
Finite-time maximum Lyapunov exponent (LE) under different control parameters.

Based on equation (3.22), the finite-time maximum LE was calculated under different control parameters. As shown in figure 4, the smoothed LE remains positive over most of the tested parameter interval, with 96.7% of the sampled points located in the positive-LE region. This result indicates that the proposed chaotic system maintains sensitive dependence on initial conditions across a broad parameter range. Although the estimated LE values are relatively small, the predominance of positive values confirms the existence of sustained chaotic behaviour.

The grey curve represents the raw finite-time LE, the blue curve represents the smoothed LE trend and the shaded region indicates the positive-LE interval. The smoothed LE remains positive over 96.7% of the tested parameter points.

When the LE is positive, small perturbations in the initial state are amplified during iteration, making long-term prediction difficult. In a spatio-temporal coupled lattice, the chaotic intensity must be evaluated over the entire lattice rather than at a single site. Therefore, the positive part of the local LE is retained for each lattice site,

(3.27) $$\lambda^{+}(i,j)=\{\begin{array}{ccc} & \lambda (i,j),\lambda (i,j)>0 & \\ & 0,\lambda (i,j)\leq 0 & \end{array}.$$

The KS entropy density is then obtained by averaging the positive local LE over the lattice,

(3.28) $$h=\frac{1}{R\cdot L}\sum_{i=1}^{R}\sum_{j=1}^{L}\lambda^{+}(i,j).$$

A larger KS entropy density indicates a higher information-generation rate and stronger unpredictability. If the entropy density approaches zero over a wide parameter region, the system is probably dominated by stable, periodic or weakly chaotic evolution.

**Figure 5.**
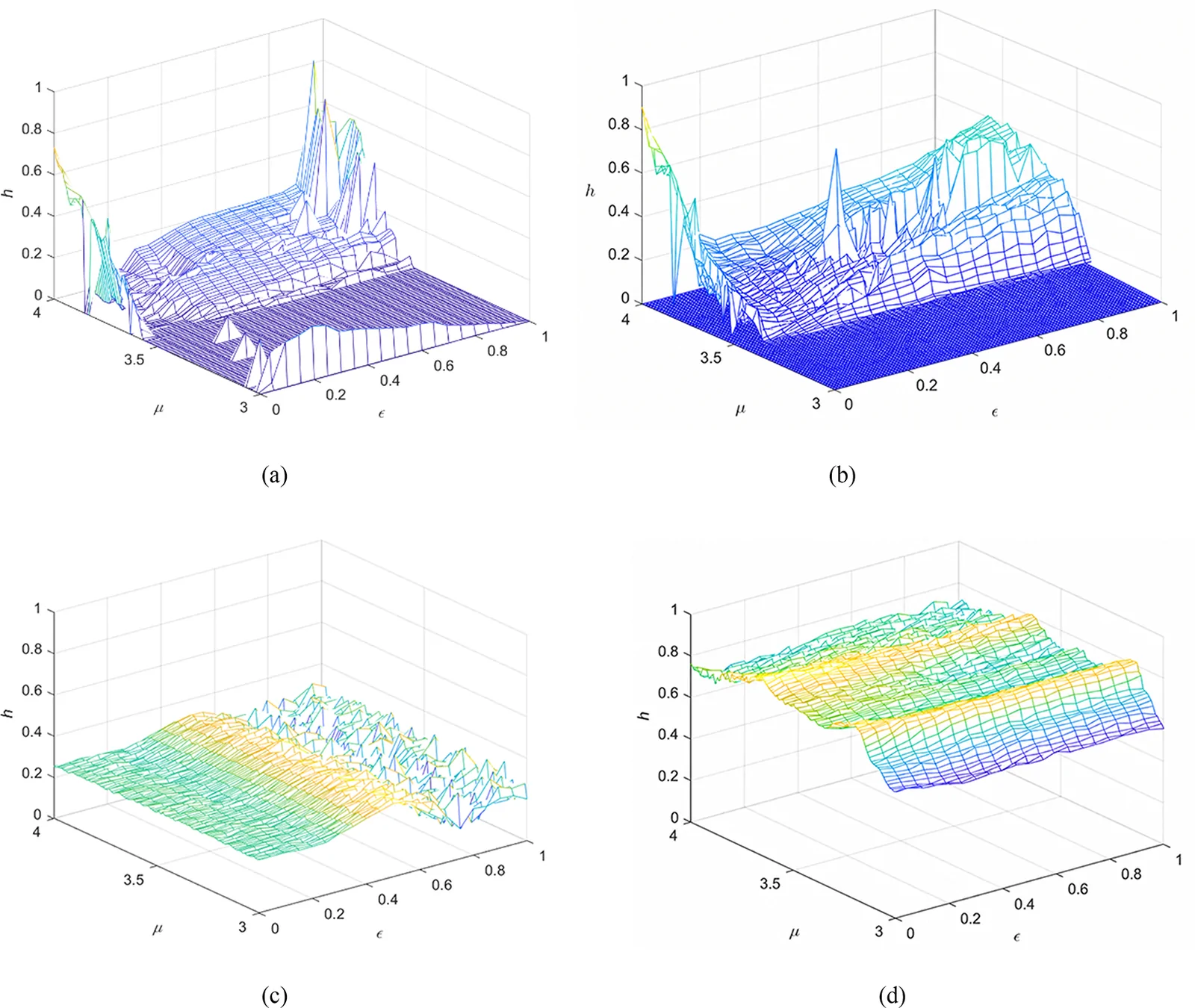
Kolmogorov–Sinai (KS) entropy density of (a) two-dimensional CML, (b) two-dimensional NLCML, (c) MC-HDCES, and (d) the proposed system.

Figure 5 compares the KS entropy density surfaces of the tested systems. The original two-dimensional CML shows large low-entropy regions and sharp isolated peaks, which means that strong chaos appears only in limited parameter intervals. The two-dimensional NLCML reduces part of the low-entropy area, but the surface still contains clear valleys and abrupt fluctuations. The memory-conditioned Hopfield-driven chaotic evolution system (MC-HDCES)/MCPML model further smooths the entropy surface and expands the effective chaotic region, although non-uniform entropy distribution is still present.

The proposed system forms a broad high-entropy plateau over nearly the entire parameter plane. Low-entropy voids and isolated extreme peaks are largely suppressed, indicating that the system maintains strong and stable chaotic behaviour under a wider range of parameters. This result supports the suitability of the proposed chaotic source for encryption, because a wider high-entropy region corresponds to a larger usable key space and stronger resistance to parameter-induced degradation.

#### 3.4.3. Correlation analysis

Correlation analysis was conducted to examine the temporal and spatial dependence of the generated chaotic sequences. In chaotic sequence generation, lower correlation means faster memory loss, weaker predictability and stronger statistical independence among outputs. For coupled map lattice systems, cross-correlation among lattice sites is particularly important because highly correlated sites may generate redundant keystreams and reduce cryptographic security.

**Figure 6.**
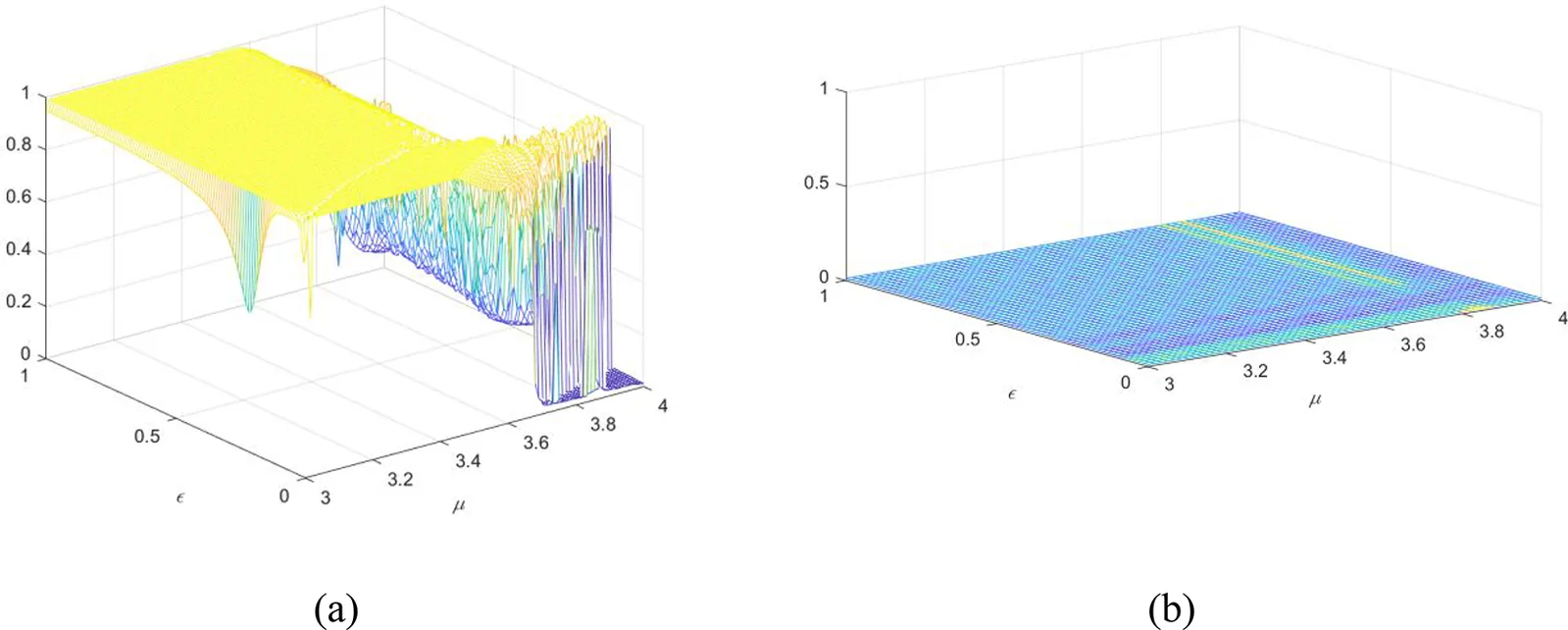
(a) Correlation analysis of the two-dimensional CML; (b) correlation analysis of the system proposed in this work.

Figure 6 compares the correlation characteristics of the original two-dimensional CML and the proposed system. In the original model, the mean cross-correlation remains high over most parameter settings. Although the correlation decreases in a narrow parameter band, most regions still exhibit strong dependence among lattice sites. This means that the original system can generate nearly independent outputs only under limited parameter conditions.

In the proposed system, the correlation surface remains close to zero across almost the entire parameter plane. Only small random fluctuations are observed, and no broad high-correlation region appears. This demonstrates that the proposed ECA-driven adaptive coupling and feedback modulation effectively weaken the dependence among lattice states. As a result, the generated sequences have better independence and are more suitable for use as cryptographic driving sequences.

#### 3.4.4. National Institute of Standards and Technology tests

The randomness of the generated binary keystream was evaluated using the National Institute of Standards and Technology (NIST) statistical test suite (STS). The NIST STS contains 15 statistical tests that examine different randomness properties, including frequency balance, run distribution, spectral characteristics, approximate entropy, serial complexity and random excursions. The significance level was set to α = 0.01. In the experiment, multiple binary sequences generated by the proposed chaotic system were submitted to the test suite.

For each test, two criteria were considered. First, the pass proportion must be higher than the theoretical lower bound,

(3.29) $$LB=(1-\alpha )-3\sqrt{\frac{\alpha (1-\alpha )}{M}}.$$

For *M* = 100 and *α* = 0.01, the lower bound is approximately 0.96015. Second, the distribution of *p*-values must be approximately uniform in [0,1]. This was checked by dividing the *p*-values into 10 intervals and applying a chi-square goodness-of-fit test to obtain the second-level *p*-value.

As shown in table 1, the binary sequences generated by the proposed chaotic system pass all NIST SP 800-22 randomness tests. For the tests evaluated over 100 sequences, the pass proportions range from 97/100 to 100/100, satisfying the required pass-rate criterion. In particular, the frequency, cumulative sums, runs, fast Fourier transform (FFT) and overlapping template tests achieve 99/100, while the block frequency, longest run, rank, non-overlapping template, serial and linear complexity tests reach 100/100. The universal and approximate entropy tests also obtain acceptable pass proportions of 97/100. For the random excursions and random excursions variant tests, only 66 sequences meet the validity conditions, and both tests achieve a pass proportion of 66/66. Moreover, all second-level *p*-values are greater than 0.01, with the minimum value being 0.01265 in the linear complexity test. These results indicate that the generated binary sequences exhibit satisfactory statistical randomness and are suitable for cryptographic keystream generation in the proposed image encryption scheme.

**Table 1.** NIST test.

statistical test	C1	C2	C3	C4	C5	C6	C7	C8	C9	C10	*p*-value	proportion
**frequency**	9	9	9	7	16	7	15	8	16	4	0.07117	99/100
**block frequency**	4	10	8	9	11	12	13	16	7	10	0.35048	100/100
**cumulative sums**	8	14	9	10	11	9	10	14	9	6	0.77918	99/100
**runs**	10	5	10	9	12	15	15	8	11	5	0.27570	99/100
**longest run**	8	7	8	13	14	9	14	8	5	14	0.31908	100/100
**rank**	7	11	16	10	10	8	6	14	10	8	0.47498	100/100
**FFT**	9	16	11	13	7	11	10	7	10	6	0.51412	99/100
**non-overlapping template**	9	11	11	10	12	9	10	10	8	10	0.99882	100/100
**overlapping template**	13	13	9	12	10	8	13	6	7	9	0.71974	99/100
**universal**	10	8	7	8	13	9	10	9	8	18	0.38382	97/100
**approximate entropy**	9	13	15	11	8	11	9	12	5	7	0.53414	97/100
**random excursions**	8	8	4	7	7	8	5	3	8	8	0.73991	66/66
**random excursions variant**	5	9	9	7	6	6	5	7	6	6	0.93195	66/66
**serial**	8	6	8	10	12	10	11	11	11	13	0.91141	100/100
**linear complexity**	18	6	8	6	15	6	4	14	14	9	0.01265	100/100

## 4. Image encryption system

After the key- and session-dependent dynamic chaotic S-box is generated, the proposed encryption system integrates TV-BST row–column permutation, S-box-based bidirectional diffusion and HDTCML-based block key mixing into a unified colour image encryption framework. The TV-BST module generates key-dependent row and column permutation indices to disrupt the spatial adjacency of image pixels. The optimized S-box provides nonlinear substitution during diffusion, while the HDTCML mechanism introduces plaintext digest, random nonce, block feedback and block-specific key mixing into the encryption process. As a result, the cipher image is jointly determined by the secret key, plaintext content, block position, channel index and local feedback context.

### 4.1. Overall architecture of the proposed cipher

The proposed cipher is a permutation-substitution-diffusion framework for colour image encryption. Its input is a colour plain image *I*, a secret key *K* and a session nonce *v*. The output is the cipher image C, together with the metadata required for decryption, including the session nonce and image-size information.

Let

(4.1) $$I\in {\left\{0,1,\ldots ,255\right\}}^{M\times N\times 3},$$

where *M* and *N* are the image height and width. If the image size is not divisible by the block size *B*, padding is applied to obtain *I_p_*, and the original size is stored for decryption.

Before encryption, the proposed chaotic system generates a chaotic sequence Ω. Based on *K*, *v* and the chaotic sequence, a chaos-driven dynamic finite-field dual-affine S-box and its inverse are generated as

(4.2) $$\left(S_{K,v},S_{K,v}^{-1}\right)=G_{S}\left(K,v,\Omega \right).$$

The detailed S-box construction is given in §4.4.

For the *r*th round, the TV-BST module first performs key-dependent row–column permutation,

(4.3) $$X^{\left(r\right)}=P_{r}\left(X^{\left(r-1\right)};K,v\right),\;\;\;X^{\left(0\right)}=I_{p}.$$

The permuted image is then divided into non-overlapping *B* × *B* blocks. For the *q*th block in channel *c*, the HDTCML module generates two block-specific diffusion key streams,

(4.4) $$\left(K_{r,q,c}^{f},K_{r,q,c}^{b}\right)=G_{K}\left(K,v,r,q,c,D,F\right),$$

where *D* is the plaintext digest and *F* is the feedback state.

Using the dynamic S-box and the two key streams, the bidirectional diffusion module encrypts each block as

(4.5) $$C_{r,q,c}=D_{S}\left(X_{r,q,c},K_{r,q,c}^{f},K_{r,q,c}^{b},S_{K,v}\right).$$

After all blocks and channels are processed, the cipher blocks are reassembled. After *R* rounds, the final cipher image is

(4.6) $$C=X^{\left(R\right)}.$$

Thus, the architecture contains four coupled modules: TV-BST row–column permutation, chaos-driven dynamic finite-field dual-affine S-box generation, HDTCML block key mixing and S-box-based bidirectional diffusion. Decryption regenerates the same dynamic S-box, inverse S-box, permutation indices and diffusion key streams from the secret key and metadata to reverse the process exactly.

### 4.2. Time-varying bit-switching transform-based row–column permutation

After preprocessing, the padded image is first subjected to TV-BST-based row–column permutation. This stage aims to disrupt the spatial adjacency of image pixels before block-wise key mixing and value diffusion are performed. Unlike a fixed permutation rule, the permutation indices are generated from a round-dependent key, so that the spatial scrambling pattern varies with the secret key, the session nonce and the encryption round.

For the *r*th encryption round, the permutation key is constructed as

(4.7) $$K_{r}=K∥v∥r,$$

where *K* is the secret key, *v* is the session nonce, *r* is the round index and ∥ denotes concatenation. The TV-BST process uses *K_r_* to generate two pseudo-random chaotic sequences,

(4.8)x(r)={x1,x2,⋯,xMp},(4.9)andy(r)={y1,y2,⋯,yNp},

where *M_p_* and *N_p_* are the height and width of the padded image, respectively. The row and column permutation indices are then obtained by sorting these two sequences,

(4.10)πr=argsortx(r),(4.11)andσr=argsorty(r).

Here, π_*r*_ denotes the row permutation index, and σ_*r*_ denotes the column permutation index. Using these indices, the image is permuted along both spatial dimensions. Let *I*^(*r* − 1)^ denote the input image of the *r*th round. The permuted image *P*^(*r*)^ is defined as

$$\begin{array}{r} P^{\left(r\right)}(i,j,c)=I^{\left(r-1\right)}\left(\pi_{r}(i),\sigma_{r}(j),c\right), \end{array}$$

where *i* = 1, 2, …, *M_p_*, *j* = 1, 2, …, *N_p_* and *c* = 1, 2, …, *C*. For RGB images, the same row and column permutation indices are applied to all three colour channels. This strategy preserves the alignment among the R, G and B channels while destroying the spatial adjacency of pixels within each channel.

The TV-BST permutation stage provides the spatial confusion component of the proposed cipher. Since the permutation depends on *K*, *v* and *r*, different encryption sessions or different rounds produce different row-column scrambling patterns. This reduces the correlation between neighbouring pixels before the subsequent HDTCML-based key mixing and S-box bidirectional diffusion stages.

### 4.3. Hopfield-driven time-varying coupled map lattice-based block key mixing

After TV-BST row–column permutation, the permuted image is divided into non-overlapping blocks. This block-wise structure allows the encryption system to generate local diffusion keys for different image regions, rather than applying a single global keystream to the whole image. Let the permuted image in the *r*th round be denoted as *P*^(*r*)^. It is partitioned into *Q* blocks,

(4.12) $$P^{\left(r\right)}=\left\{P_{1}^{\left(r\right)},P_{2}^{\left(r\right)},\ldots ,P_{Q}^{\left(r\right)}\right\}.$$

For the *b*th block and the *c*th colour channel, a block-specific context is constructed by combining the secret key, session nonce, plaintext digest, feedback state, round index, block index and channel index,

(4.13) $$\Omega_{r,b,c}=K\|v\|D\|F_{r,b}\|r\|b\|c,$$

where *K* denotes the secret key, *v* denotes the session nonce, *D* denotes the plaintext digest, *F*_*k*,*b*_ denotes the feedback state used for the current block, *r* denotes the round index, *b* denotes the block index, *c* denotes the channel index and ∥ denotes concatenation. Through this construction, each block is encrypted under a different local context, even when the same image and the same secret key are used.

The context Ω_*r*,*b*, *c*_ is then used to drive the HDTCML process and generate two block-level key components,

(4.14) $$\left(H_{f}^{r,b,c},H_{b}^{r,b,c}\right)=H_{HDTML}(\Omega_{r,b,c}),$$

where $H_{f}^{r,b,c}$ and $H_{b}^{r,b,c}$ represent the forward and backward HDTCML-derived key components, respectively. These two components correspond to the two scan directions used in the subsequent bidirectional diffusion stage.

In parallel, a TV-BST byte-level keystream is generated for the same block and channel,

(4.15) $$K_{TVBST}^{r,b,c}=T_{TVBST}(K,v,r,b,c).$$

This keystream is divided into forward and backward parts,

(4.16) $$K_{TVBST}^{r,b,c}=\left(K_{T,f}^{r,b,c},K_{T,b}^{r,b,c}\right).$$

The final diffusion keys are obtained by exclusive OR (XOR) mixing the TV-BST keystream with the HDTCML-derived key components,

(4.17)Kfr,b,c=KT,fr,b,c⊕Hfr,b,c,(4.18)andKbr,b,c=KT,br,b,c⊕Hbr,b,c,

where ⊕ denotes bitwise XOR. Therefore, the forward and backward diffusion keys are not generated by a single chaotic source. Instead, they are jointly determined by the TV-BST keystream and the HDTCML block context.

The feedback state *F*_*r*,*b*_ controls how ciphertext information participates in later block encryption. In the general form, it can be written as

(4.19) $$F_{r,b}=\{\begin{array}{cllc} & Reset(K,v,D,r,b), & (b-1)\;\mathrm{mod}\;\rho =0, & \\ & SHA256(C_{r,b-1}), & otherwise, & \end{array}$$

where ρ denotes the feedback reset period and *C*_*r*,*b* − 1_ denotes the previous cipher block. This mechanism allows the encryption context to incorporate local ciphertext feedback while still controlling the range of error propagation. In the crop-robust configuration used in this work, ρ = 1, so the feedback state is reset for every block,

(4.20) $$F_{r,b}=Reset(K,v,D,r,b).$$

This setting makes different blocks more independent and reduces the possibility that a cropped or corrupted cipher block contaminates the key generation of subsequent blocks. Thus, HDTCML-based block key mixing provides both block-level key diversity and a controllable mechanism for limiting error propagation under ciphertext cropping or occlusion attacks.

### 4.4. Chaos-driven dynamic S-box generation

The nonlinear substitution layer of the proposed cipher is provided by a chaos-driven dynamic finite-field dual-affine S-box. Unlike a fixed substitution table, the proposed S-box is generated from the secret key and the chaotic sequence produced by the proposed chaotic system. Therefore, different keys or sessions can produce different substitution mappings while preserving exact reversibility.

Let the chaotic sequence used for S-box generation be denoted as

(4.21) $$\Omega =\left\{\omega_{1},\omega_{2},\ldots \right\},\;\;\;\omega_{i}\in \left(0,1\right).$$

The sequence Ω is used to control the selection of the finite field and the affine parameters. First, an irreducible polynomial *p*(*x*) of degree 8 is selected from a predefined polynomial pool,

(4.22) $$p\left(x\right)\in P=\left\{p_{1}\left(x\right),p_{2}\left(x\right),\ldots ,p_{L}\left(x\right)\right\}.$$

The selected polynomial defines the finite field *GF*(2^8^). Then, two invertible binary matrices and two offset vectors are generated,

(4.23) $$A_{1},A_{2}\in GL\left(8,2\right),\;\;\;b_{1},b_{2}\in GF{\left(2\right)}^{8},$$

where *A*_1_ and *A*_2_ are non-singular over *GF*(2). The matrix *A*_1_ and vector *b*_1_ form the input affine layer, while *A*_2_ and *b*_2_ form the output affine layer.

For an input byte *x* ∈ {0, 1, …, 255}, let its binary vector representation be

(4.24) $$x={\left(x_{0},x_{1},\ldots ,x_{7}\right)}^{T}\in GF{\left(2\right)}^{8}.$$

The input affine transformation is defined as

(4.25) $$u=A_{1}x⊕b_{1}.$$

The vector **u** is then interpreted as an element of *GF*(2^8^) under the selected irreducible polynomial *p*(*x*). A finite-field inverse operation is applied,

(4.26) $$z=Inv_{p}\left(u\right),$$

where ${\text{Inv}}_{p}(\cdot )$ denotes multiplicative inversion in *GF*(2^8^) defined by *p*(*x*), with zero mapped to zero. Finally, the output affine transformation is performed,

(4.27) $$y=A_{2}z⊕b_{2}.$$

Thus, the dynamic S-box is defined as

(4.28) $$S_{K,v}\left(x\right)=Byte\left(y\right)$$

or equivalently,

(4.29) $$S_{K,v}\left(x\right)=A_{2}\left(Inv_{p}\left(A_{1}x⊕b_{1}\right)\right)⊕b_{2}.$$

Here, *S*_*k*.*v*_ denotes the S-box generated under the secret key *K* and session nonce *v*. Since *A*_1_ and *A*_2_ are invertible and the finite-field inverse operation is bijective, the generated S-box is also bijective. Therefore, its inverse S-box can be deterministically regenerated during decryption using the same key and session parameters.

**Figure 7.**
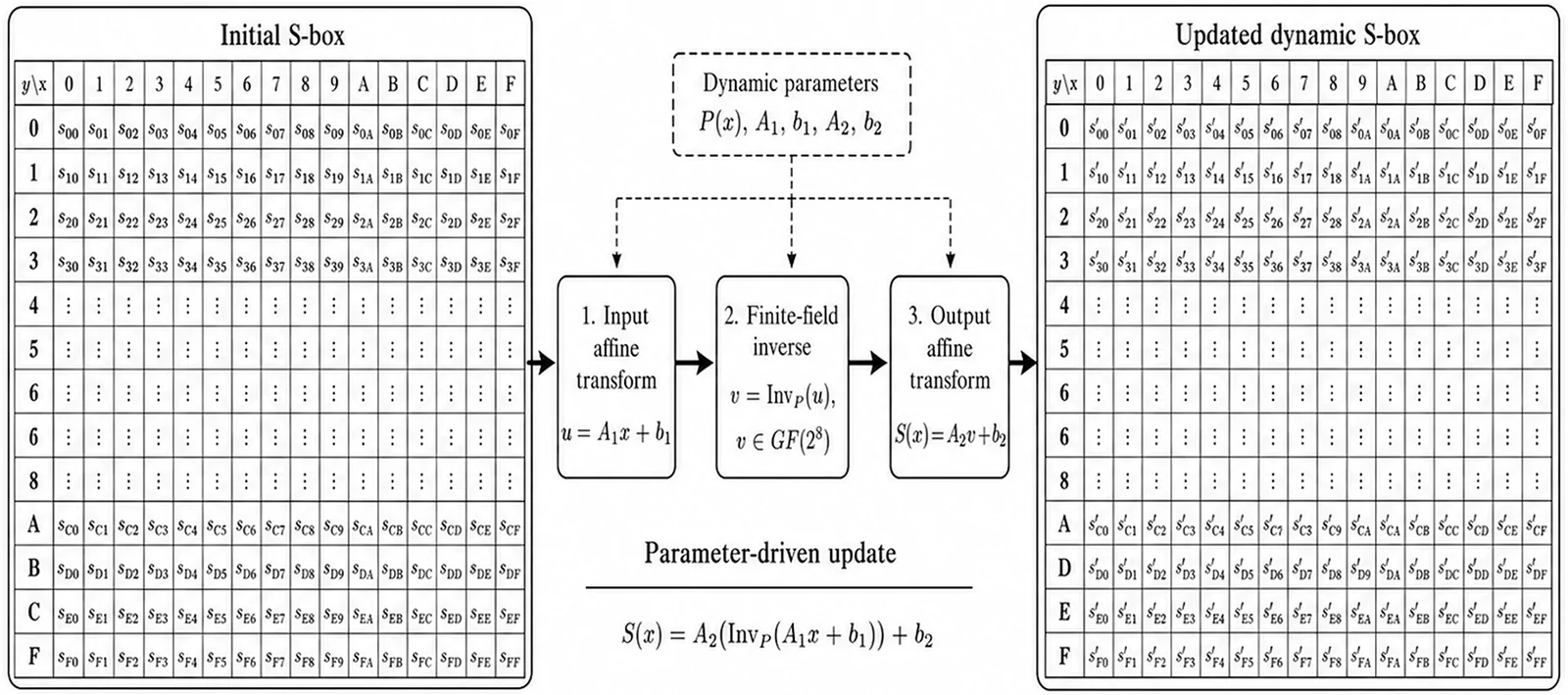
Transformation mechanism of the chaos-driven dynamic affine S-box.

The overall transformation mechanism of the proposed chaos-driven dynamic affine S-box is illustrated in figure 7.

To avoid selecting a weak substitution table, the proposed method generates a candidate pool of dynamic S-boxes. For each candidate, the chaotic sequence determines a polynomial *p*(*x*), two affine matrices *A*_1_, *A*_2_ and two offsets *b*_1_, *b*_2_. Each candidate S-box is evaluated using cryptographic indicators, including nonlinearity, differential uniformity, linear approximation probability, strict avalanche criterion, fixed points, reverse fixed points and short cycles.

The candidate score is defined as

(4.30) $$F=NL_{avg}+NL_{min}-DU-\lambda_{1}\Delta_{SAC}-\lambda_{2}N_{FP}-\lambda_{3}N_{RFP}-\lambda_{4}N_{SC},$$

where *NL*_avg_ and *NL*_min_ denote the average and minimum nonlinearity, *DU* is the differential uniformity, Δ_*SAC*_ is the strict avalanche criterion (SAC) deviation and *N_FP_*, *N_RFP_* and *N_SC_* denote the numbers of fixed points, reverse fixed points and short cycles, respectively.

A candidate is regarded as safe only if it satisfies the following constraints:

(4.31) $$NL_{min}\geq 112,\;\;\;DU\leq 4,\;\;\;N_{FP}=0,\;\;\;N_{RFP}=0.$$

If multiple safe candidates are obtained, the final S-box is selected from the top-scoring candidates under the joint control of the secret key and chaotic sequence. This strategy keeps the S-box generation key-dependent and dynamic, while ensuring that the selected substitution table satisfies the required cryptographic properties.

The resulting *S*_*k*,*v*_ is used in the bidirectional diffusion module described in §4.5. During encryption, it provides nonlinear substitution for pixel values. During decryption, the inverse table $S_{k,v}^{-1}$ is used to exactly recover the original values.

### 4.5. Dynamic S-box-based bidirectional diffusion

After TV-BST permutation and HDTCML-based block key mixing, each permuted image block is encrypted by S-box-based bidirectional diffusion. This stage combines the regenerated chaos-driven dynamic finite-field dual-affine S-box from §4.4 with the block-specific forward and backward key streams generated in §4.3.

For the *q*th block in channel *c* during the *r*th round, let the permuted block be denoted as

(4.32) $$X_{r,q,c}\in {\left\{0,1,\ldots ,255\right\}}^{B\times B}.$$

It is converted into a one-dimensional sequence according to the predefined scan order,

(4.33) $$P=\left\{p_{1},p_{2},\ldots ,p_{L}\right\},\;\;\;L=B^{2}.$$

The corresponding forward and backward key streams are denoted as

(4.34) $$\begin{array}{clc} & K_{r,q,c}^{f}=\left\{k_{1}^{f},k_{2}^{f},\ldots ,k_{L}^{f}\right\} & \\ & and\;\;K_{r,q,c}^{b}=\left\{k_{1}^{b},k_{2}^{b},\ldots ,k_{L}^{b}\right\} & \end{array}.$$

The forward diffusion pass scans the sequence from the first position to the last position. Let *F* = {*f*_1_,*f*_2_,…, *f_L_*} be the intermediate sequence after forward diffusion. The forward process is defined as

(4.35) $$f_{i}=S_{K,v}\left(p_{i}⊕k_{i}^{f}⊕f_{i-1}\right),\;\;\;i=1,2,\ldots ,L$$

with the initial condition

(4.36) $$f_{0}=0.$$

The backward diffusion pass further scans the intermediate sequence in reverse order. Let *C* = {*c*_1_,*c*_2_,…, *c_L_*} be the final cipher sequence. The backward process is defined as

(4.37) $$c_{i}=S_{K,v}\left(f_{i}⊕k_{i}^{b}⊕c_{i+1}\right),\;\;\;i=L,L-1,\ldots ,1$$

with the boundary condition

(4.38) $$c_{L+1}=0.$$

The final cipher block is obtained by reshaping *C* back to the original block size,

(4.39) $$C_{r,q,c}=reshape\left(C,B,B\right).$$

In this structure, the forward pass propagates changes from earlier positions to later positions, while the backward pass propagates dependencies from later positions to earlier positions. The dynamic S-box *S*_*k*,*v*_ introduces nonlinear substitution in both passes. Therefore, each cipher byte is jointly determined by the permuted input byte, the forward and backward diffusion keys, the chained neighbouring state and the key-dependent dynamic S-box.

Since *S*_*k*,*v*_ is bijective and *XOR* is self-inverse, the bidirectional diffusion process can be exactly reversed during decryption using the inverse S-box $S_{k.v}^{-1}$ and the same regenerated key streams. The inverse process is described in §4.6.

### 4.6. Reversible decryption

The proposed TVBST-HDTCML encryption process is fully reversible because each operation in the encryption path has a deterministic inverse. During decryption, the receiver uses the same secret key *K* and metadata to regenerate the dynamic S-box, its inverse S-box, the TV-BST permutation indices and the block-level diffusion key streams.

For the *q*th cipher block in channel *c* during the *r*th round, let the cipher sequence be

(4.40) $$c_{r,q,c}=\{c_{1},c_{2},\ldots ,c_{L}\},$$

where *L* = *B* × *B*. Decryption first reverses the backward diffusion pass. Using the regenerated backward key stream $k_{r,q,c}^{b}$, the intermediate sequence is recovered as

(4.41) $$u_{i}=S_{K}^{-1}(c_{i})⊕k_{r,q,c}^{b}(i)⊕c_{i+1},\;\;\;i=L,L-1,\ldots ,1$$

with the boundary condition

(4.42) $$c_{L+1}=0.$$

After obtaining the intermediate sequence {*u*_1_,*u*_2_,…, *u_L_*}, the forward diffusion pass is inverted using the regenerated forward key stream $k_{r,q,c}^{f}$,

(4.43) $$p_{i}=S_{K}^{-1}(u_{i})⊕k_{r,q,c}^{f}(i)⊕u_{i-1},\;\;\;i=1,2,\ldots ,L$$

with

(4.44) $$\mu_{0}=0.$$

The recovered sequence

(4.45) $$p_{r,q,c}=\{p_{1},p_{2},\ldots ,p_{L}\}$$

is then reshaped into the recovered permuted block

(4.46) $$P_{r,q,c}\in Z_{256}^{B\times B}.$$

After all blocks and colour channels are processed, the recovered blocks are reassembled to form the permuted image *P*^(*r*)^. The inverse TV-BST row–column permutation is then applied to recover the image before the *r*th encryption round

(4.47) $$I_{p}^{\left(r-1\right)}=P_{r}^{-1}\left(P^{\left(r\right)};K,v\right).$$

For a multi-round setting, the above inverse operations are performed from round *R* to round 1. After all rounds are reversed, the padded plain image $I_{p}^{}$ is obtained. Finally, the padding region is removed according to the original image size recorded in the metadata, yielding the recovered image

(4.48) $$\hat{I}=Unpad(I_{p},M,N).$$

Since the regenerated S-box is bijective, XOR is self-inverse, the TV-BST permutation has deterministic inverse indices and the block-level key streams can be regenerated from the same key and metadata, the proposed encryption system supports exact decryption when the correct key and metadata are provided. This reversibility also ensures that the dynamic S-box introduced in §4.4 does not affect the lossless recovery of the plain image.

## 5. Experimental results and security analysis

**Figure 8.**
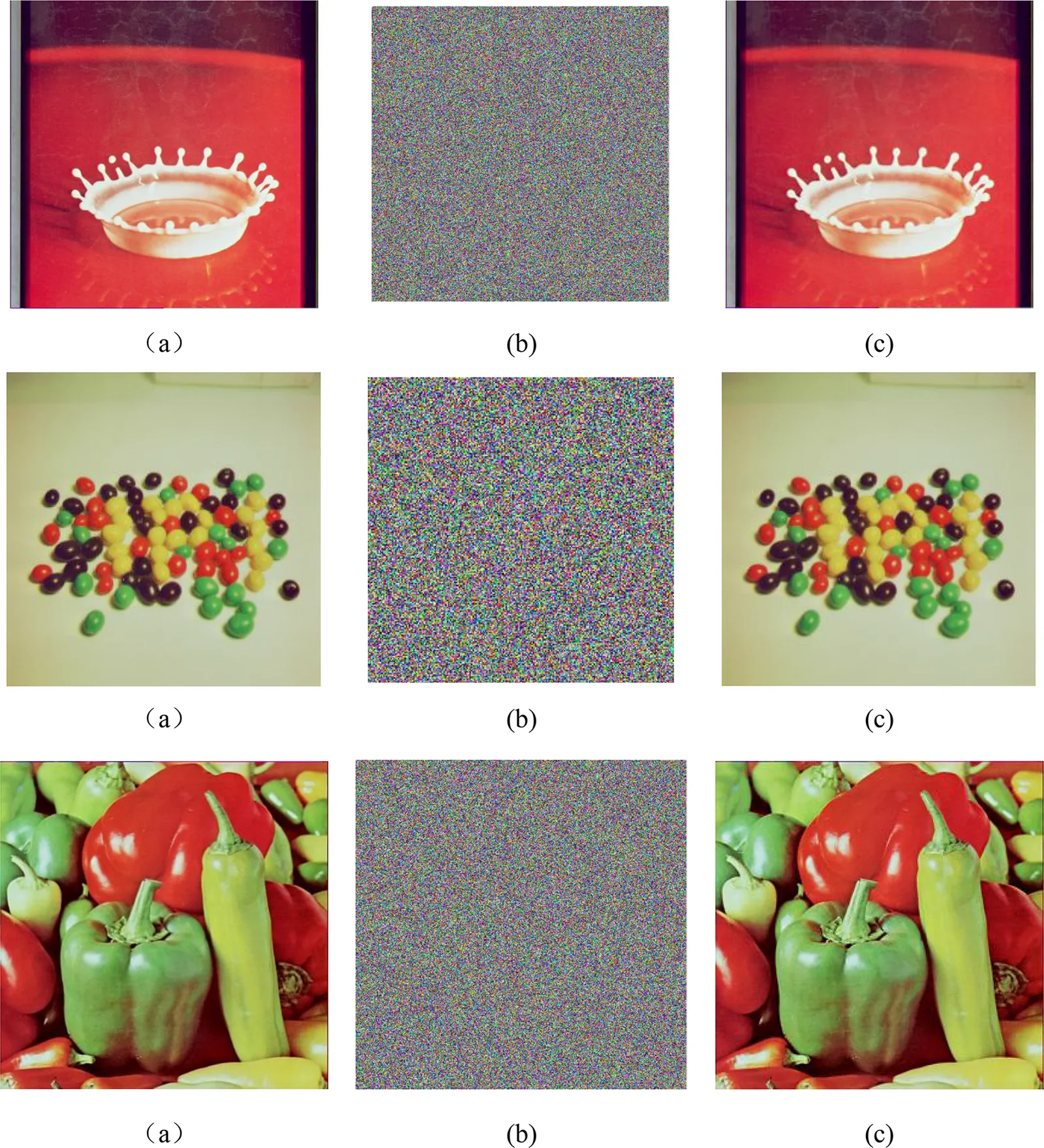
Encryption and decryption results for splash, jelly beans and peppers: (a) plaintext image, (b) encrypted image and (c) decrypted image.

The security performance of the proposed image encryption scheme was evaluated from five aspects: dynamic S-box performance, key-space and key-sensitivity analysis, statistical security, differential attack resistance and robustness against ciphertext degradation. The experiments were conducted on standard colour images, including splash, jelly beans and peppers. For each test image, encryption and decryption were performed using the proposed TVBST-HDTCML framework with the chaos-controlled dynamic S-box  *S_K_*. The corresponding plain images, cipher images and decrypted images are shown in figure 8.

### 5.1. Dynamic S-box performance analysis

Since the dynamic S-box *S_K_* is the nonlinear substitution component of the proposed cipher, its cryptographic properties were evaluated before the complete image-encryption experiments. The generated S-box was tested in terms of bijectivity, nonlinearity, differential approximation probability, linear approximation probability, strict avalanche criterion, bit independence criterion and the corresponding differential distribution table (DDT), LAT and SAC distributions. The results show that *S_K_* satisfies bijectivity and exhibits strong nonlinear substitution capability, low differential and linear approximation probabilities and balanced avalanche behaviour. These properties indicate that the proposed dynamic S-box can effectively enhance the confusion performance of the encryption scheme.

A valid 8-bit S-box should be a permutation of {0, 1, …, 255}, which guarantees the existence of the inverse S-box $\;S_{{}_{K}}^{-1}$. The test results confirmed that the generated  *S_K_* is bijective. Therefore, the substitution operation used in encryption can be exactly reversed during decryption.

The nonlinearity and differential properties were further calculated to evaluate the resistance of  *S_K_* against linear and differential cryptanalysis. As reported in table 2, the generated S-box achieved stable cryptographic indicators, including high nonlinearity, low differential uniformity and low linear approximation probability. In addition, the SAC and BIC results indicate that small changes in the S-box input can produce balanced changes in the output bits.

**Table 2.** Cryptographic performance comparison of the proposed dynamic S-box.

	nonlinearity					SAC				
S-Box	min	max	avg	DAP	LAP	min	max	avg	BIC-SAC	BIC-NL
AES	112	112	112	0.0156	0.0620	0.3671	0.5975	0.5040	0.5020	112
proposed S-Box	112	112	112	0.0156	0.0625	0.4375	0.5625	0.5081	0.5001	112
[30]	110	112	110.75	0.0234	0.0859	0.4375	0.5625	0.4976	0.5034	110.07

**Figure 9.**
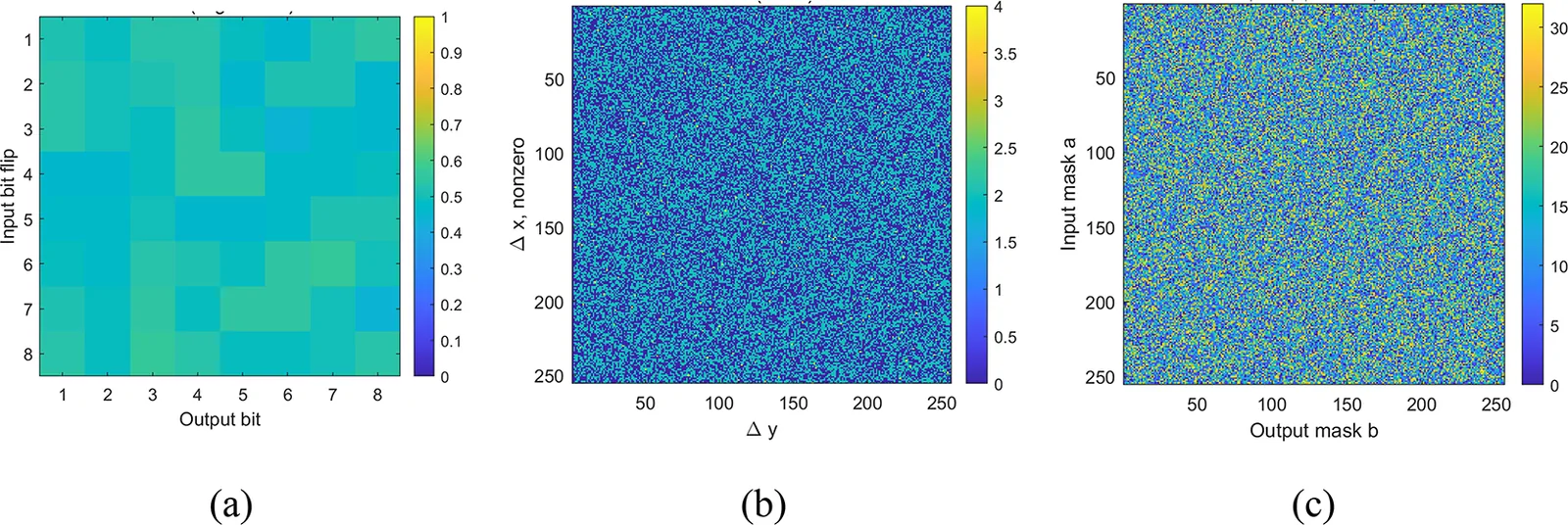
SAC, DDT and LAT heat maps of the proposed dynamic S-box: (a) SAC matrix, (b) DDT excluding zero input difference and (c) absolute LAT excluding zero masks.

The comparative results in table 2 further demonstrate the cryptographic strength of the proposed dynamic S-box. The proposed S-box achieves a minimum, maximum and average nonlinearity of 112, which is identical to the AES S-box and higher than [30], whose average nonlinearity is 110.75. In terms of differential and linear resistance, the proposed S-box obtains differential approximation probability (DAP) = 0.0156 and linear approximation probability (LAP) = 0.0625, matching the strong differential behaviour of AES and clearly outperforming [30], which gives DAP = 0.0234 and LAP = 0.0859. For the SAC property, the proposed S-box has values ranging from 0.4375 to 0.5625, with an average value of 0.5081, indicating that its output-bit changes remain close to the ideal probability of 0.5 under one-bit input perturbations. Moreover, the BIC-SAC value of 0.5001 and bit independence criterion–nonlinearity (BIC-NL) value of 112 show that the output bits maintain good independence and high nonlinear behaviour. Overall, these results indicate that the proposed dynamic S-box provides strong nonlinearity, low differential and linear approximation probabilities, satisfactory avalanche behaviour and competitive or superior cryptographic performance compared with AES and [30]. As shown in figure 9 and table 3, the proposed dynamic S-box exhibits SAC values close to the ideal value of 0.5, while the DDT and LAT heat maps further illustrate its differential and linear distribution characteristics.

**Table 3.** SAC matrix of the proposed dynamic S-box.

0.48438	0.48438	0.51562	0.50000	0.45312	0.53125	0.57812	0.53125
0.51562	0.51562	0.48438	0.48438	0.59375	0.48438	0.50000	0.48438
0.51562	0.50000	0.51562	0.48438	0.53125	0.54688	0.45312	0.51562
0.51562	0.51562	0.50000	0.49875	0.51562	0.48750	0.46875	0.45312
0.53125	0.46875	0.48438	0.48438	0.46875	0.50000	0.43750	0.46875
0.56250	0.51562	0.46875	0.50000	0.50000	0.46875	0.50938	0.51562
0.48438	0.49750	0.50000	0.53125	0.51562	0.40625	0.54688	0.50250
0.51562	0.48438	0.50000	0.53125	0.53125	0.48438	0.54688	0.51562

### 5.2. Key space and key sensitivity analysis

A secure image cipher requires a sufficiently large key space and strong sensitivity to key perturbations. The former determines the resistance against exhaustive search, whereas the latter ensures that an approximate key cannot reproduce a meaningful encryption or decryption result.

In the proposed scheme, the secret key *K* is used not only to initialize the chaotic source, but also to derive the dynamic S-box, TV-BST permutation indices and HDTCML-based block diffusion keys. Therefore, a small variation in *K* affects multiple encryption stages simultaneously. Assuming that a 256-bit secret key is used, the effective key space is

(5.1) $$K=2^{256}.$$

This value is much larger than the commonly accepted security threshold of 2^100^, indicating that brute-force search is computationally infeasible.

To evaluate key sensitivity, the same plain image was encrypted using two keys *K* and *K'*, where *K'* differs from *K* by only one bit. The corresponding cipher images are denoted as

(5.2) $$C=E(I,K),\;\;C^{\prime }=E(I,K^{\prime }).$$

The structural similarity between *C* and *C'* was measured by structural similarity index measure (SSIM). A value close to zero indicates that the two cipher images share little structural similarity. As shown in table 4, the cipher images generated under the two slightly different keys exhibit very low SSIM values, confirming the key sensitivity of the proposed encryption scheme.

(5.3) $$\text{SSIM}(x,y)=\frac{2\mu_{x}\mu_{y}+C_{1}}{\mu_{x}^{2}+\mu_{y}^{2}+C_{1}}\cdot \frac{2\sigma_{x}\sigma_{y}+C_{2}}{\sigma_{x}^{2}+\sigma_{y}^{2}+C_{2}}\cdot \frac{\sigma_{xy}+C_{3}}{\sigma_{x}\sigma_{y}+C_{3}}.$$

**Table 4.** SSIM values of the encrypted images.

image	SSIM
splash	0.005583
jelly beans	0.0066707
peppers	0.0079426
tree	0.0078469
sailboat	0.0066491

### 5.3. Statistical security analysis

Statistical security was evaluated by histogram distribution, adjacent-pixel correlation and information entropy. These metrics examine whether the cipher image still preserves distributional or structural traces of the plain image.

#### 5.3.1. Histogram analysis

Natural colour images usually have non-uniform RGB-channel histograms because pixel intensities are related to image content, texture and illumination. An effective encryption scheme should flatten these distributions and produce cipher images with approximately uniform intensity histograms.

**Figure 10.**
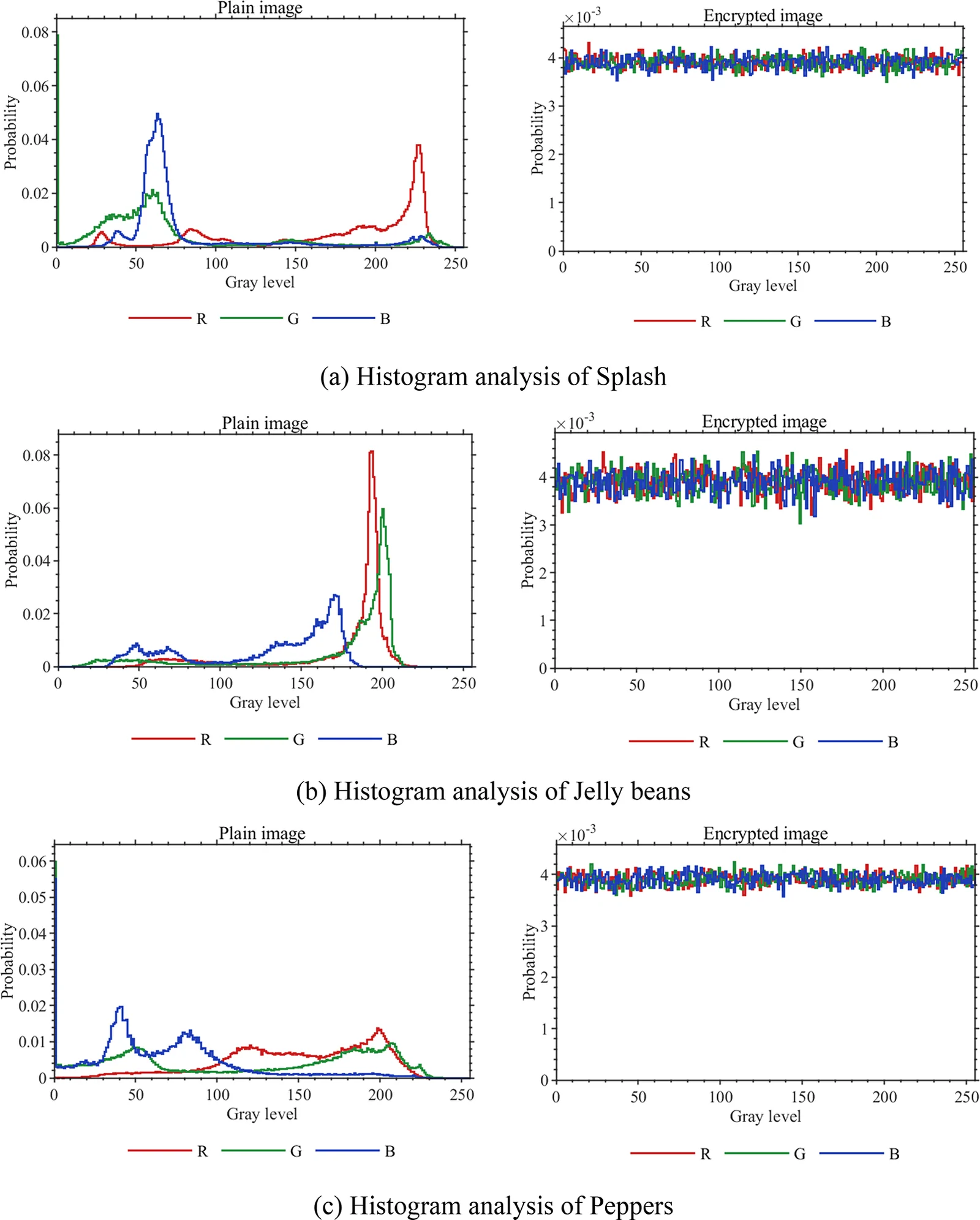
RGB-channel histograms of plain and cipher images: (a) splash, (b) jelly beans and (c) peppers.

Figure 10 shows the RGB-channel histograms of splash, jelly beans and peppers before and after encryption. The plain images exhibit distinct peaks and channel-dependent distributions, whereas the cipher images show nearly uniform histograms over the whole grey-level range. This result indicates that the proposed encryption process effectively conceals the statistical distribution of the original image.

To avoid relying only on visual observation, the histogram variance (HV) was further introduced to quantitatively evaluate the uniformity of the encrypted histograms. For an 8-bit colour image, let *h_i_* denote the number of pixels at grey level *i*, where *i* = 0, 1, …, 255, and let *h̄* = *MN*/256 denote the ideal average frequency of each grey level for an image of size *M* × *N*. The HV is calculated as the mean squared deviation between *h_i_* and *h̄*. A smaller HV value indicates that the histogram is closer to the ideal uniform distribution.

(5.4) $$HV=\frac{1}{256}\sum_{i=0}^{255}{\left(h_{i}-\bar{h}\right)}^{2}.$$

The quantitative results are listed in table 5. It can be observed that the plain images have large HV values because their pixel distributions are strongly related to image contents, textures and illumination. By contrast, the encrypted images exhibit much smaller HV values in the R, G and B channels. This demonstrates that the proposed encryption algorithm effectively flattens the pixel distribution and makes the cipher histograms close to a uniform distribution. Therefore, the statistical characteristics of the original images are concealed, which improves the resistance of the proposed scheme against histogram-based statistical attacks.

**Table 5.** Comparison of histogram variance (HV) and reduction ratio before and after encryption.

image	plain image HV	encrypted image HV	reduction ratio (%)
splash	3 807 837.21	1003.75	99.9736
jelly beans	338 837.91	251.07	99.9259
peppers	1 389 272.56	738.12	99.9469

#### 5.3.2. Adjacent pixel correlation analysis

Adjacent-pixel correlation was used to evaluate whether local spatial dependence remains after encryption. For a plain image, neighbouring pixels in the horizontal, vertical and diagonal directions usually show high correlation. The correlation coefficient is defined as

(5.5) $$\rho_{xy}=\frac{E[(x-E(x))(y-E(y))]}{\sqrt{D(x)}\sqrt{D(y)}},$$

where *x* and *y* are the grey values of two adjacent pixels, and *E*( · ) and *D*( · ) denote expectation and variance, respectively.

**Figure 11.**
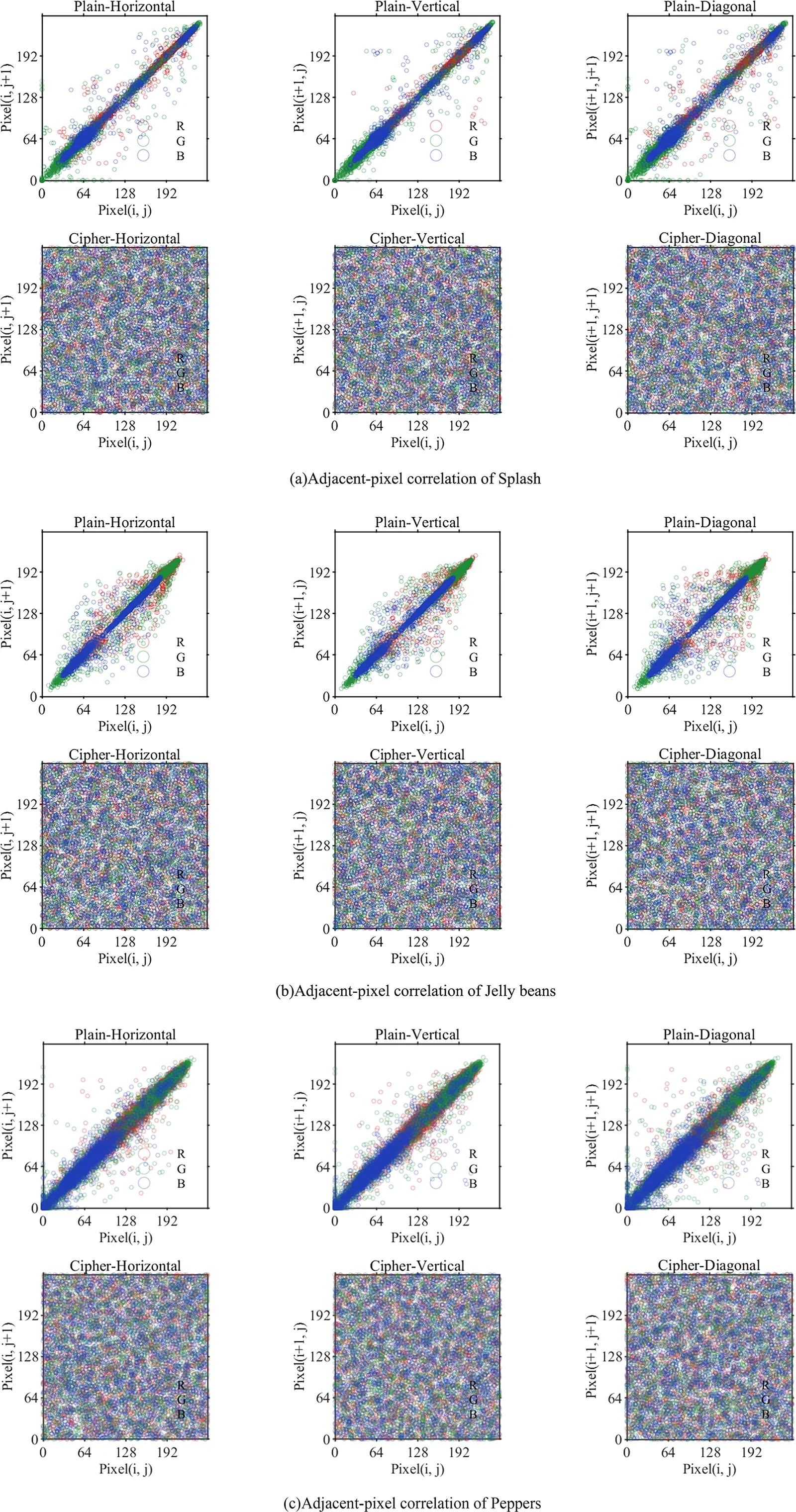
Correlation analysis results for splash, jelly beans and peppers. (*Continued overleaf.*)

As shown in figure 11, the plain images have high adjacent-pixel correlations, whereas the cipher images have correlation coefficients close to zero in all three directions. The scatter plots also change from diagonal concentration to random-like dispersion. These results show that the proposed permutation–diffusion structure effectively suppresses local structural leakage.

#### 5.3.3. Information entropy analysis

Information entropy was used to quantify the uncertainty of the encrypted images. For an 8-bit image, the ideal entropy value is 8. The entropy is defined as

(5.6) $$H(I)=-\sum_{i=0}^{255}p(i){\text{log}}_{2}p(i),$$

where *p*(*i*) denotes the probability of grey value *i*. For colour images, entropy was calculated for each RGB channel, and the average value was used as the final result.

As reported in table 6, the entropy values of the cipher images are close to the theoretical maximum of 8. This indicates that the encrypted images have high uncertainty and low grey-level predictability. Together with the histogram and correlation results, the entropy analysis confirms that the proposed scheme effectively suppresses statistical information in cipher images.

**Table 6.** Information entropy of the encrypted images.

image	plain image	cipher image
splash	6.653	7.9993
jelly beans	6.27	7.9990
peppers	7.297	7.9997

### 5.4. Differential attack analysis

Differential attack analysis was performed to evaluate the sensitivity of the proposed scheme to slight plaintext perturbations. Two plain images *I*_1_ and *I*_2_ were used, where *I*_2_ differs from *I*_1_ by only one pixel. The corresponding cipher images are denoted as

(5.7) $$C_{1}=E(I_{1},K),\;\;\;C_{2}=E(I_{2},K).$$

The NPCR and UACI were used to quantify the difference between *C*_1_ and *C*_2_. They are defined as

(5.8) $$\text{NPCR}=\frac{1}{MNC}\sum_{i=1}^{M}\sum_{j=1}^{N}\sum_{k=1}^{C}D(i,j,k)\times 100\%,$$

where

(5.9) $$D(i,j,k)=\{\begin{array}{cllc} & 0, & C_{1}(i,j,k)=C_{2}(i,j,k), & \\ & 1, & C_{1}(i,j,k)\neq C_{2}(i,j,k), & \end{array}$$

and

(5.10) $$\text{UACI}=\frac{1}{MNC}\sum_{i=1}^{M}\sum_{j=1}^{N}\sum_{k=1}^{C}\frac{\left|C_{1}(i,j,k)-C_{2}(i,j,k)\right|}{255}\times 100\%.$$

As shown in table 7, the obtained NPCR and UACI values are close to their theoretical reference values for 8-bit images. For the three test images, the NPCR values remain around 99.6094% in all RGB channels, with values ranging from 99.5853% to 99.6429%. Meanwhile, the UACI values are close to the expected value of 33.4635%, ranging from 33.3950% to 33.5869%. These results indicate that a one-pixel perturbation in the plain image can be effectively propagated to the cipher image across different colour channels. Therefore, the proposed encryption scheme exhibits strong differential sensitivity and provides effective resistance against differential attacks.

**Table 7.** UACI and NPCR before and after encryption for different images.

	UACI			NPCR		
image	R	G	B	R	G	B
splash	33.4341%	33.4131%	33.4319%	99.6273%	99.6155%	99.6082%
jelly beans	33.5249%	33.3950%	33.4349%	99.6429%	99.5926%	99.5972%
peppers	33.5475%	33.5869%	33.4341%	99.6292%	99.5853%	99.6181%

Table 8 compares the UACI and NPCR values of the encrypted peppers image obtained by different methods. The proposed method achieves UACI values of 33.5475%, 33.5869% and 33.4341% for the R, G and B channels, respectively, which are close to the ideal value of 33.4635%. Meanwhile, the corresponding NPCR values are 99.6292%, 99.5853% and 99.6181%, remaining close to the theoretical expectation of 99.6094%. Compared with the referenced methods, the proposed scheme maintains more balanced UACI performance across the three colour channels and achieves competitive NPCR results, indicating that a slight change in the plaintext can be effectively propagated throughout the ciphertext image.

**Table 8.** UACI and NPCR of peppers after encryption using different methods.

	UACI			NPCR		
image	R	G	B	R	G	B
this article	33.5475%	33.5869%	33.4341%	99.6292%	99.5853%	99.6181%
[31]	33.4655%	33.4781%	33.4746%	99.6137%	99.6137%	99.6137%
[32]	33.4280%	33.4966%	33.3779%	99.6052%	99.6052%	99.6052%
[33]	33.6770%	28.8684%	24.4740%	99.6070%	99.6070%	99.6070%
[34]	33.2450%	33.3620%	33.5210%	99.6230%	99.6230%	99.6230%

### 5.5. Computational complexity and scalability analysis

In addition to security performance, computational efficiency was also evaluated. For an RGB image with size H × W, let *N* = H × W denote the number of pixels. The proposed scheme mainly consists of chaotic sequence generation, TV-BST-based row–column permutation, dynamic S-box substitution and bidirectional diffusion. Since these operations are mainly local, pixel-wise or traversal-based, the overall computational complexity of the encryption and decryption processes is O(*N*), and the memory complexity is also O(*N*).

Execution-time tests were conducted on resized images, and the results are reported in table 9.

**Table 9.** Execution time and scalability evaluation of the proposed encryption scheme.

image size	number of pixels	mean encryption time (s)	std. time (s)	throughput (MPixels s^–1^)
128 × 128	16 384	1.1563	0.2450	0.0142
256 × 256	65 536	3.6106	0.0793	0.0182

As shown in table 9, when the image size increases from 128 × 128 to 256 × 256, the number of pixels increases fourfold, while the encryption time increases from 1.1563 to 3.6106 s. This indicates that the implementation cost grows in a controlled manner with image size. Although the current MATLAB implementation has not yet been optimized for real-time deployment, the local update, pixel-wise substitution, permutation and diffusion operations are suitable for further acceleration through vectorization, parallel computation or hardware implementation.

### 5.6. Robustness against data loss and noise attacks

To evaluate robustness against ciphertext degradation, cropping and salt-and-pepper noise attacks were applied to the encrypted images before decryption.

**Figure 12.**
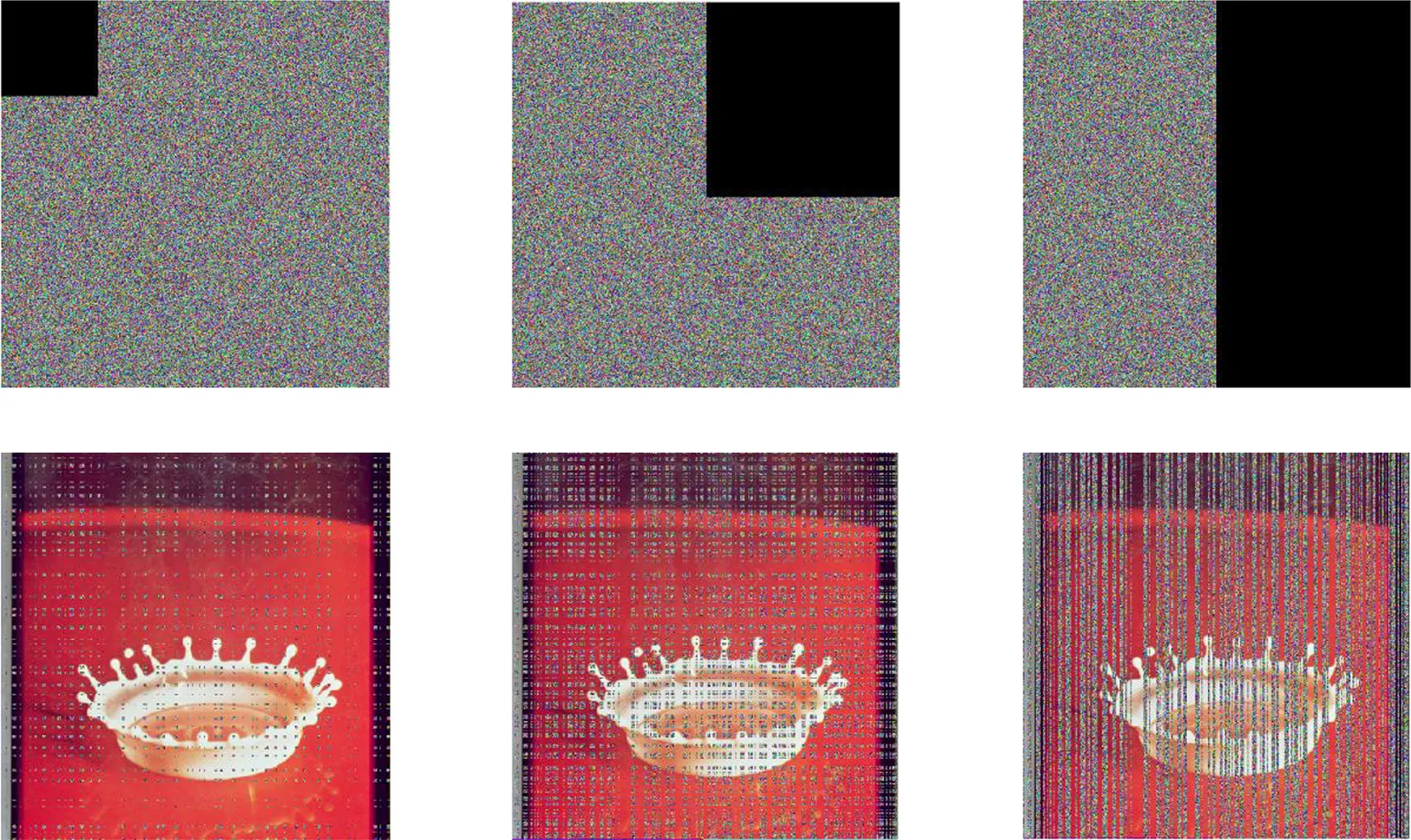
Robustness analysis against cropping attacks.

As shown in figure 12, the cipher images were cropped with ratios of 0.0625, 0.25 and 0.5. With a small cropping ratio, the decrypted image remains visually clear. As the cropped area increases, more distortion and stripe-like artefacts appear, but the main image content is still recognizable even under 0.5 cropping. This indicates that the proposed scheme can limit error propagation caused by partial ciphertext loss.

**Figure 13.**
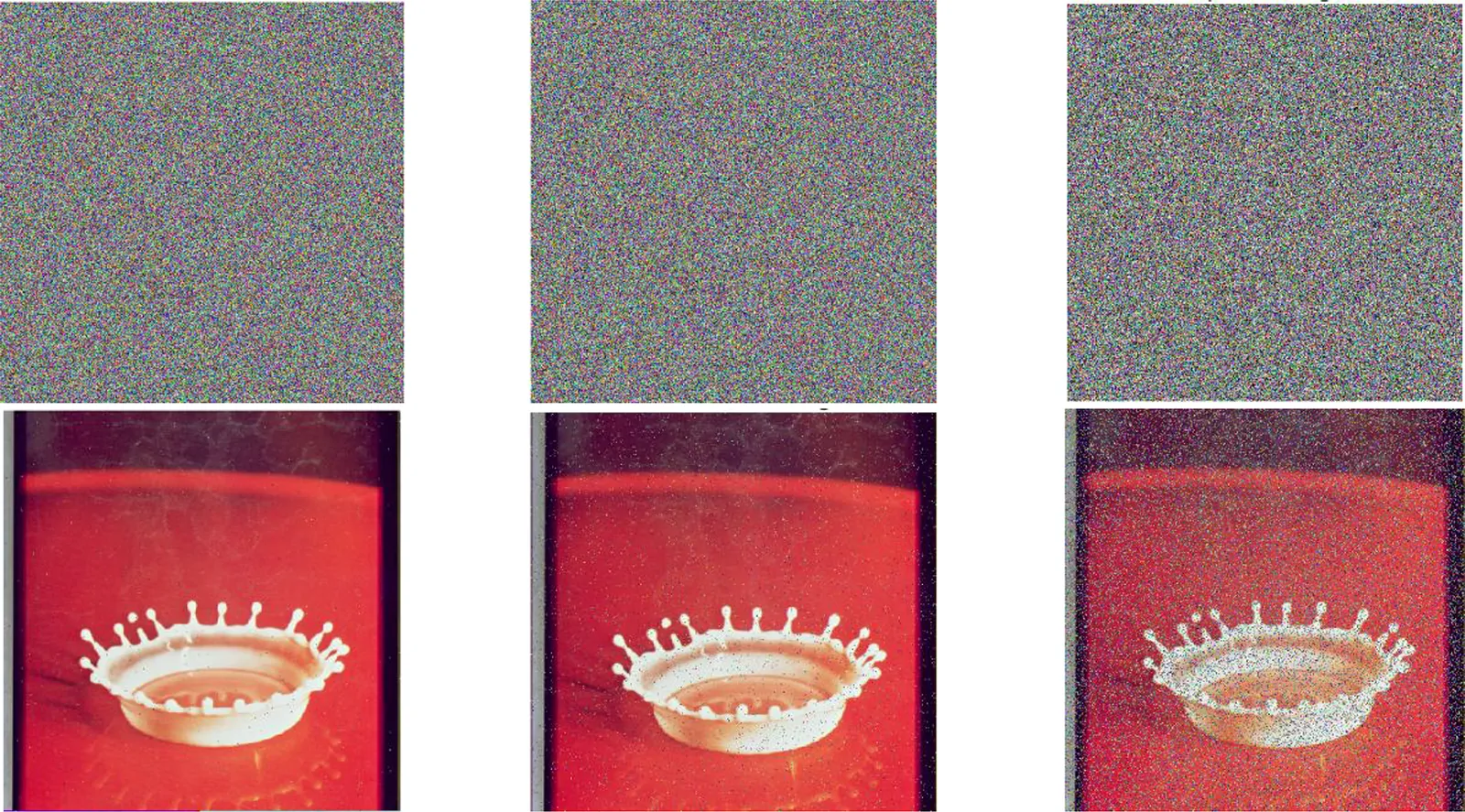
Robustness analysis under salt-and-pepper noise attacks.

Figure 13 shows the results under salt-and-pepper noise with densities of 0.001, 0.01 and 0.1. The recovered image is only slightly affected at low noise density, while visible noise points increase as the density becomes larger. Even at density 0.1, the main object can still be identified. These results show that the proposed method has a certain robustness against noise contamination, although visual quality decreases under stronger attacks.

Overall, the results in figures 12 and 13 demonstrate that the proposed encryption scheme can preserve recognizable image content under moderate cropping and noise attacks.

### 5.7. Chosen-plaintext attack analysis

Chosen-plaintext attacks represent a stronger threat model in which an attacker can deliberately select plaintext images and observe their corresponding ciphertexts. To evaluate the resistance of the proposed scheme under this scenario, five highly structured plaintext images were selected, including all-zero, all-255, checquerboard, horizontal-gradient and structured-ramp images. These plaintexts contain simple or regular intensity distributions and are, therefore, suitable for testing whether the encryption process leaks plaintext structure into the ciphertext.

The ciphertexts generated from these chosen plaintexts were evaluated using information entropy and adjacent-pixel correlation coefficients in the horizontal, vertical and diagonal directions. The results are shown in table 10.

**Table 10.** Ciphertext statistical results under chosen-plaintext attack.

chosen plaintext	entropy	corr-H	corr-V	corr-D
all-zero plaintext	7.999282	0.002421	0.002056	0.002564
all-255 plaintext	7.999277	0.001956	0.001169	0.002002
checquerboard plaintext	7.999309	0.001169	0.001565	0.001407
horizontal-gradient plaintext	7.999254	0.001942	0.001482	0.000609
structured-ramp plaintext	7.999310	0.002659	0.000525	0.001879

As shown in table 10, all ciphertexts generated from the selected structured plaintexts have entropy values close to the ideal value of 8. In addition, the horizontal, vertical and diagonal adjacent-pixel correlation coefficients are all close to zero. These results indicate that even when the plaintext image has an extremely simple or highly regular structure, the corresponding ciphertext still exhibits random-like statistical characteristics. Therefore, the proposed encryption scheme can effectively conceal plaintext structure and shows resistance to chosen-plaintext attacks.

## 6. Conclusion

This article presents a colour image encryption method that jointly designs adaptive spatio-temporal chaos, dynamic finite-field dual-affine S-box construction and coordinated permutation–diffusion. By replacing fixed lattice evolution with state-dependent regulation, the proposed chaotic model improves sequence complexity, sensitivity and randomness. The generated chaotic flow is further embedded into dynamic S-box construction, strengthening nonlinear substitution and differential resistance.

The encryption framework integrates TV-BST permutation, HDTCML block key mixing and dynamic S-box-based bidirectional diffusion. Experimental results show that the scheme generates random-like ciphertexts, suppresses adjacent-pixel correlation, improves plaintext sensitivity and resists statistical, differential, noise, cropping and chosen-plaintext attacks. These findings indicate that coupling chaotic dynamics with substitution and diffusion design can improve the security of colour image encryption.

Future work will focus on reducing computational cost for lightweight and embedded deployment. Further studies will also extend the framework to video and real-time multi-media encryption, while evaluating its robustness under broader cryptanalytic and hardware implementation scenarios.

## Data Availability

The standard test images used and analysed in this study were obtained from the Miscellaneous Volume of the USC-SIPI Image Database, maintained by the Signal and Image Processing Institute, University of Southern California, and are publicly available at https://sipi.usc.edu/database/database.php?volume=misc (accessed 19 April 2026).
